# One-Pot Synthesis of Triazolobenzodiazepines Through Decarboxylative [3 + 2] Cycloaddition of Nonstabilized Azomethine Ylides and Cu-Free Click Reactions

**DOI:** 10.3390/molecules24030601

**Published:** 2019-02-08

**Authors:** Xiaoming Ma, Xiaofeng Zhang, Weiqi Qiu, Wensheng Zhang, Bruce Wan, Jason Evans, Wei Zhang

**Affiliations:** 1 School of Pharmaceutical Engineering and Life Science, Changzhou University, Changzhou 213164, China; mxm.wuxi@cczu.edu.cn; 2 Department of Chemistry, University of Massachusetts Boston, 100 Morrissey Boulevard, Boston, MA 02125, USA; xfxiaofengzhang@gmail.com (X.Z.); Weiqi.Qiu001@umb.edu (W.Q.); brucewan99@yahoo.com (B.W.); jason.evans@umb.edu (J.E.); 3 School of Science, Jiaozuo Teachers’ College, 998 Shanyang Road, Jiaozuo 454100, China; tongjizws@163.com

**Keywords:** one-pot synthesis, decarboxylative [3 + 2] cycloaddition, nonstabilized azomethine ylides, click reaction

## Abstract

A one-pot synthesis of triazolobenzodiazepine-containing polycyclic compounds is introduced. The reaction process involves a decarboxylative three-component [3 + 2] cycloaddition of nonstabilized azomethine ylides, *N*-propargylation, and intramolecular click reactions.

## 1. Introduction

**Figure 1:**
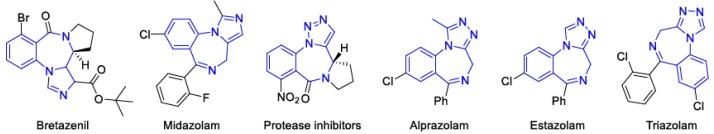
Biologically active triazolobenzodiazepine-related molecules.

Triazolobenzodiazepines and related scaffolds are privileged heterocyclic systems for biologically active molecules, such as benzodiazepine-bearing bretazenil [1], midazolam [2]; protease inhibitors [3], alprazolam [4], estazolam [5], and triazolam [6] (Figure 1). Due to their medicinal significance, the development of synthetic methods for triazolobenzodiazepine-bearing compounds continuously attracts the attention of organic and medicinal chemists [7,8,9].

**Scheme 1:**
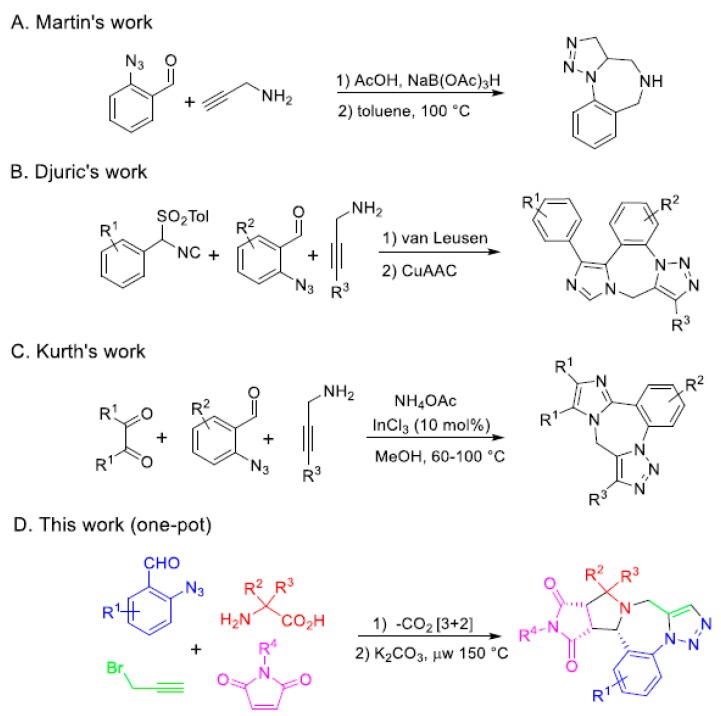
Atom economic synthesis of triazolobenzodiazepines.

Highly efficient and atom economic synthesis such as one-pot reactions and multicomponent reactions (MCRs) have gained increasing popularity in the synthesizing of complex molecules including triazolobenzodiazepine-type compounds [10,11,12,13,14,15]. For example, the Martin group reported a cascade reductive amination and intramolecular [3 + 2] cycloaddition reaction sequence for triazole-fused 1,4-benzodiazepines (Scheme 1A) [10,11]. The Djuric group modified the van Leusen imidazole synthesis to develop an intramolecular azide-alkyne cycloaddition for imidazole- and triazole-fused benzodiazepine compounds (Scheme 1B) [12]. The Kurth group reported a Lewis acid-catalyzed MCR for imidazole- and triazole-fused benzodiazepines through sequential [3 + 2] cycloaddition and cycloaddition reactions (Scheme 1C) [13]. Introduced in this paper is a new sequence involving decarboxylative intermolecular [3 + 2] cycloaddition of nonstabilized azomethine ylides followed by *N*-propargylation and intramolecular [3 + 2] cycloaddition for triazolobenzodiazepines (Scheme 1D).

**Figure 2:**
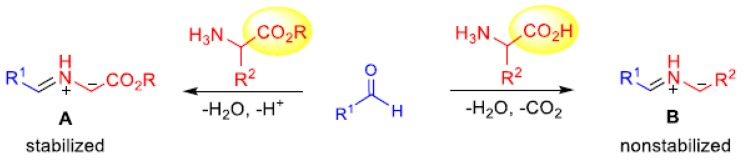
Azomethine ylides from amino esters or amino acid.

1,3-Dipolar cycloaddition of primary amino esters, aldehydes, and activated alkenes is a well-established three-component reaction [16,17,18,19,20,21]. The azomethine ylides derived from deprotonation of iminium ions are CO_2_R-stabilized ylides A (Figure 2A) [22,23,24,25,26,27,28,29,30]. In recent years, our lab has reported a series of azomethine ylides A-based [3 + 2] cycloadditions for diverse heterocyclic scaffolds [31,32,33,34,35], including one-pot [3 + 2] and click reactions for triazolobenzodiazepines [32]. Compared to the reactions of stabilized ylides A, cycloadditions of nonstabilized ylides B are less explored (Figure 2B) [36,37,38,39,40,41,42]. We have recently reported the synthesis of α-trifluoromethyl pyrrolidines through decarboxylative [3 + 2] cycloaddition of nonstabilized azomethine ylides B derived from α-amino acids [43]. Presented in this paper is a new application of nonstabilized azomethine ylides in the one-pot [3 + 2] and click reactions for triazolobenzodiazepines.

## 2. Results and Discussions

Reaction conditions for the synthesis of proline **4a** through one-pot [3 + 2] cycloaddition were developed using 1:1.2:1 of 2-azidebenzaldehyde **1a**, 2-aminoisobutyric acid **2a,** and *N*-ethylmaleimide **3a** in the presence of 0.3 equiv. of AcOH for decarboxylation [43] (Table 1). After screening solvents including 2-methyltetrahydrofuran, toluene, EtOH and CH_3_CN as well as reaction time and temperature, it was found that a reaction using CH_3_CN as a solvent at 110 °C for 6 h afforded **4a** in 93% LC (liquid chromatography) yield with a dr (diastereomer) of 6:1 (Table 1, entry 6). The stereochemistry of **4a** was determined according to the literature report [38].

Decarboxylative [3 + 2] cycloaddition product **4a** was then used for the development of conditions for the *N*-propargylation and sequential click reaction for the synthesis of triazolobenzodiazepine **6a**. In the presence of K_2_CO_3_, **4a** reacted with propargyl bromide in CH_3_CN at 80 °C for 2 h to give **5a** in 94% LC yield (Table 2, entries 2–5). Without separation, the reaction mixture was used for intramolecular click reaction at 100 °C under the catalysis of Cu salts (Table 2, entries 2–4). The CuI-catalyzed click reaction gave **6a** in 89% LC yield, which is better than the reactions catalyzed with CuCl or CuBr. In our previous work, the intramolecular click reaction was accomplished under microwave heating and Cu-free conditions [32]. In this work, *N*-propargylation compound **5a** generated under the microwave heating was continuously heated at 150 °C for 1 h to give **6a** in 88% LC yield without CuI catalyst (Table 2, entry 6). A Cu-free control reaction of **5a** under conventional heating at 100 °C for 3 h only gave 5% of **6a** (Table 2, entry 5).

After establishing the three-component [3 + 2] cycloaddition, *N*-propargylation, and sequential click reactions for **6a** shown in Table 1 and Table 2, we then aimed to combine these three reactions in one pot. After modification of the conditions shown in Table 1 and Table 2, the best conditions for the one-pot synthesis was to conduct the decarboxylative [3 + 2] cycloaddition in MeCN under conventional heating at 110 °C for 6 h, then to perform the *N*-propargylation and spontaneous Cu-free click reaction under microwave heating at 150 °C for 1 h to give **6a** in 76% LC yield (Table 3, entry 3). A control reaction using CuI as a catalyst for the click reaction didn’t give a better yield (Table 3, entry 4).

Under the optimized conditions for the one-pot synthesis [44], 13 analogues of triazolobenzodiazepines **6a–m** were synthesized using different sets of azidobenzaldehydes **1** (R^1^ = H, CF_3_, Br, Cl, NO_2_), amino acids **2** (R^2^ = H, Me; R^3^ = Me, Ph, *i*-Pr), and maleimides **3** (R^4^ = Me, Et, Ph, Bn, 4-Br-Ph) (Table 4). The reactions of five different maleimides with 2-aminoisobutyric acids and 2-azidebenzaldehyde gave **6a–e** in 55–65% isolated yields. The substitution groups on the benzaldehydes had some influence on the product yield. For example, the azidobenzaldehydes bearing electron-withdrawing groups, such as Br and CF_3_, gave **6f** and **6g** in lower yields (59% and 35%), while the azidobenzaldehyde with the strong electron-withdrawing group NO_2_ gave no product of **6m**. The reactions of glycine and leucine with azidobenzaldehydes (R^1^ = H, Br, Cl) and maleimides (R^4^ = Me, Et) gave **6h–l** in 44–55% yields. The stereochemistry of product **6** was established during the step of the decarboxylative [3 + 2] cycloaddition, which was determined according to the literature report [38].

**Scheme 2:**
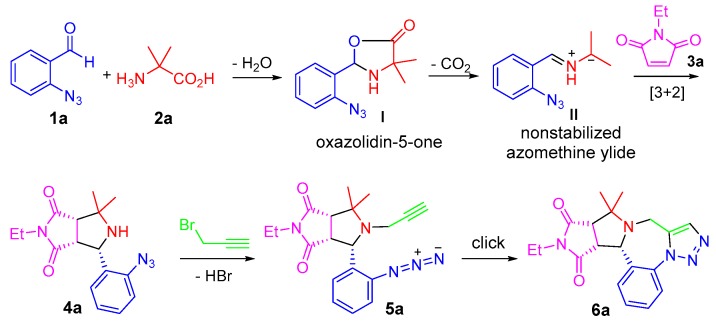
Mechanism for one-pot synthesis of **6a**.

The proposed mechanism for the synthesis of product **6a** is outlined in Scheme 2. The condensation of 2-azidebenzaldehyde **1a** and 2-aminoisobutyric acid **2a** give oxazolidin-5-one **I**. It then underwent decarboxylation to form the nonstabilized azomethine ylide **II** for [3 + 2] cycloaddition with **3a** to form **4a**. Formation of **5a** through propargylation followed by continuous heating for intramolecular click reaction affords product **6a**. There are several reports in literature which demonstrated that intramolecular click reactions in one-pot synthesis could be achieved under Cu-free conditions [10,15,32,45,46].

## 3. Summary

A one-pot synthesis of fused-triazolobenzodiazepines was developed using readily available amino acids, maleimides, and 2-azidebenzaldehydes for decarboxylative [3 + 2] cycloaddition of nonstabilized azomethine ylides, followed by *N*-propargylation and a Cu-free intramolecular click reaction. This is a highly efficient and operational simple reaction process for fused-triazolobenzodiazepines, and only CO_2_ and H_2_O were generated as byproducts.

## Figures and Tables

**Table 1:**
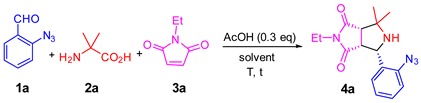
Three-component decarboxylative [3 + 2] cycloaddition ^a^.

ntry	Solvent	T (°C)	t (h)	4a (%) ^b^	dr (%) ^c^
1	2-Me THF	80	4	trace	—
2	MePh	110	4	trace	—
3	EtOH	80	4	82	5:1
4	EtOH	110	6	93	6:1
5	CH_3_CN	110	4	92	6:1
**6**	**CH_3_CN**	**110**	**6**	**93**	**6:1**
7	CH_3_CN	125	12	88	6:1

^a^ Reaction conditions: 1:1.2:1 **1a**:**2a**:**3a** for [3 + 2] cycloaddition. ^b^ Detected by LC-MS. ^c^ Determined by ^1^H NMR.

**Table 2:**
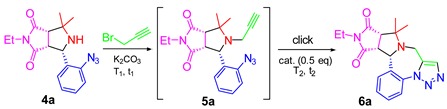
One-pot *N*-propargylation and click reaction ^a^.

Entry	Solvent	T_1_ (°C)	t_1_ (h)	5a (%) ^b^	Cat.	T_2_ (°C)	t_2_ (h)	6a (%) ^b^
1	EtOH	80	2	trace				
2	CH_3_CN	80	2	94	CuCl	100	3	35
3	CH_3_CN	80	2	94	CuBr	100	3	60
4	CH_3_CN	80	2	94	CuI	100	3	89
5	CH_3_CN	80	2	94	—	100	3	5
**6 ^c^**	**CH_3_CN**	**110**	**0.5**	**93**	**—**	**150**	**1**	**88 (dr 6:1)**

^a^ Reaction conditions: K_2_CO_3_ (2.5 equiv.) and propargyl bromide (5.0 equiv.) under conventional or microwave heating. ^b^ Detected by LC-MS. ^c^ Microwave heating for both *N*-propargylation and click reactions.

**Table 3:**
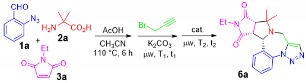
Conditions for the one-pot synthesis of 6a ^a^.

Entry	T_1_ (°C)	t_1_ (h)	Cat.	T_2_ (°C)	t_2_ (h)	6a (%) ^b^
1	110	0.5	—	150	1	75
2	150	0.5	—	150	1	51
**3**	**150**	**1**	**—**	**150**	**1**	**76 (dr 6:1)**
4	110	0.5	CuI	110	1	70

^a^ Reaction conditions: 1:1.2:1 **1a:2a:3a,** K_2_CO_3_ (2.5 equiv.), propargyl bromide (5 equiv.). ^b^ Detected by LC-MS, 6:1 dr.

**Table 4:**
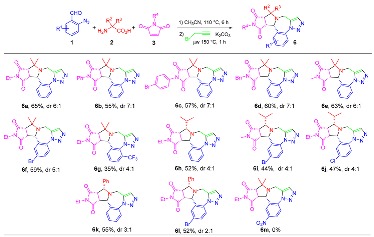
One-pot synthesis of triazolobenzodiazepines 6 ^a^.

^a^ Reaction conditions, see [44]. ^b^ Isolated yield.

## Supplementary Materials

The following are available online. ^1^H-NMR, ^13^C-NMR, and ^19^F-NMR spectra of final products.
