# Supplementary material for: One-Pot Synthesis of Triazolobenzodiazepines Through Decarboxylative [3 + 2] Cycloaddition of Nonstabilized Azomethine Ylides and Cu-Free Click Reactions
Source: Molecules. 2019 Feb 8;24(3):601. doi: 10.3390/molecules24030601 (PMC6384988; doi:10.3390/molecules24030601)

Supporting Information

One-Pot Synthesis of Triazolobenzodiazepines Through Decarboxylative [3 + 2] Cycloaddition of Nonstabilized Azomethine Ylides and Cu-Free Click Reactions

Xiaoming Ma[1], Xiaofeng Zhang[2], Weiqi Qiu[2], Wensheng Zhang[3], Bruce Wan[2], Jason Evans[2] and Wei Zhang[2]

[1] School of Pharmaceutical Engineering and Life Science, Changzhou University, Jiangsu 213164, China; E-mail: mxm.wuxi@cczu.edu.cn

[2] Department of Chemistry, University of Massachusetts Boston, 100 Morrissey Boulevard, Boston, MA 02125, USA. Fax: +1-617-287-6030; E-mail: wei2.zhang@umb.edu

[3] School of Science, Jiaozuo Teachers' College, 998 Shanyang Road, Jiaozuo 454100, China.

Contents

1. General Information ……………………………………………..…………S2

2. Analytical Data of Products …………………………………………..…….S3

3. NMR Spectra of Products ……………………………………….………S6

1. General Information

Chemicals and solvents were purchased from Siama, TCI and Oakwood. ^{1}H (400 MHz) and ^{13}C NMR spectra (101 MHz) were recorded on a 400 MHz Agilent NMR spectrometer. Chemical shifts were reported in parts per million (ppm), and the residual solvent peak was used as an internal reference: proton (chloroform δ 7.26, DMSO δ 2.50), carbon (chloroform δ 77.0, DMSO δ 39.53). Multiplicity was indicated as follows: s (singlet), d (doublet), t (triplet), q (quartet), m (multiplet), dd (doublet of doublet), br s (broad singlet). Coupling constants were reported in Hertz (Hz). LC-MS were performed on an Agilent 2100 system. A C_{18} column (5.0 μm, 6.0 x 50 mm) was used for the separation. The mobile phases were methanol and water both containing 0.05% trifluoroacetic acid. A linear gradient was used to increase from 25:75 v/v methanol/water to 100% methanol over 7.0 min at a flow rate of 0.7 mL/min. UV detections were conducted at 210 nm, 254 nm and 365 nm. Low resolution mass spectra were recorded in APCI (atmospheric pressure chemical ionization). Flash chromatography separations were performed on YAMAZEN AI-580 flash column system with Agela silica gel columns (230-400 μm mesh) and Angela Flash/Cheeta System with Venusil PrepG C_{18} column (10 μm, 120 Å, 21.2 mm x 250 mm).

1.1 General procedure for the one-pot synthesis of product 6:

A solution of 2-azidebenzaldehyde **1** (1.0 mmol), amino acid **2** (1.2 mmol) and maleimide **3** (1.0 mmol) in 3.0 mL of CH_3CN was heated at 110 °C for 6 h in a sealed tube. Upon the completion of the reaction as monitored by LC-MS, propargyl bromide solution (80% in toluene, 5.0 mmol) and K_2CO_3 (2.5 mmol) were added to the reaction mixture and then heated under microwaves at 150 °C for 1 h. The concentrated reaction mixture was isolated on a YAMAZEN AI-580 flash column or Angela Flash/Cheeta System with Venusil PrepG C_{18} column to afford purified major diastereomer of product **6**.

2. Analytical Data of Products

(11aS,14aR,14bS)-13-ethyl-11,11-dimethyl-11,11a,14a,14b-tetrahydro-9H,12H-benzo[f]pyrrolo[3',4':3,4]pyrrolo[1,2-d][1,2,3]triazolo[1,5-a][1,4]diazepine-12,14(13H)-dione ***(6a)***

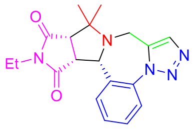

6a

White solid, 65% yield. M.P. 185–187 °C

^{1}H NMR (400 MHz, cdcl$_3$) δ 8.23 (d, *J* = 7.5 Hz, 1H), 7.62 (d, *J* = 6.2 Hz, 1H), 7.49 – 7.36 (m, 3H), 4.93 (d, *J* = 5.5 Hz, 1H), 4.01 (dd, *J* = 15.2, 2.2 Hz, 1H), 3.56 (dd, *J* = 15.2, 1.6 Hz, 1H), 3.50 – 3.44 (m, 1H), 3.35 – 3.26 (m, 2H), 2.87 (dd, *J* = 7.7, 2.3 Hz, 1H), 1.29 (d, *J* = 1.9 Hz, 3H), 1.22 (d, *J* = 2.0 Hz, 3H), 0.88 (td, *J* = 7.1, 2.3 Hz, 3H).

^{13}C NMR (100 MHz, cdcl$_3$) δ 175.50, 174.55, 136.70, 134.76, 131.27, 130.39, 128.67, 127.45, 126.43, 124.23, 67.93, 62.32, 52.99, 47.56, 37.80, 33.68, 24.63, 20.64, 12.70.

HRMS (ESI-TOF, *m/z*): [M+H]$^+$ calculated for $C_{19}H_{22}N_5O_2$ 352.1773, found 352.1774

(11aS,14aR,14bS)-11,11-dimethyl-13-phenyl-11,11a,14a,14b-tetrahydro-9H,12H-benzo[f]pyrrolo[3',4':3,4]pyrrolo[1,2-d][1,2,3]triazolo[1,5-a][1,4]diazepine-12,14(13H)-dione ***(6b)***

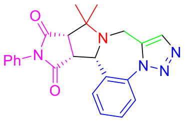

6b

White solid, 55% yield. M.P. 250–252 °C

^{1}H NMR (400 MHz, dmso) δ 8.05 – 8.00 (m, 1H), 7.74 (dd, *J* = 5.9, 3.6 Hz, 1H), 7.64 (s, 1H), 7.41 (dd, *J* = 6.1, 3.5 Hz, 2H), 7.33 (ddd, *J* = 15.7, 11.9, 7.1 Hz, 3H), 6.93 – 6.87 (m, 2H), 5.04 (d, *J* = 6.2 Hz, 1H), 4.26 (d, *J* = 15.3 Hz, 1H), 3.95 (dd, *J* = 7.7, 6.3 Hz, 1H), 3.56 (d, *J* = 15.3 Hz, 1H), 3.05 (d, *J* = 7.8 Hz, 1H), 1.21 (d, *J* = 6.6 Hz, 6H).

^{13}C NMR (101 MHz, dmso) δ 175.77, 175.41, 138.24, 134.39, 132.49, 130.95, 129.24, 128.60, 128.26, 127.58, 126.99, 123.46, 68.08, 62.65, 53.41, 48.54, 37.84, 25.15, 20.74.

HRMS (ESI-TOF, *m/z*): [M+H]$^+$ calculated for $C_{23}H_{22}N_5O_2$ 400.1773, found 400.1771

(11aS,14aR,14bS)-13-(4-bromophenyl)-11,11-dimethyl-11,11a,14a,14b-tetrahydro-9H,12H-benzo[f]pyrrolo[3',4':3,4]pyrrolo[1,2-d][1,2,3]triazolo[1,5-a][1,4]diazepine-12,14(13H)-dione ***(6c)***

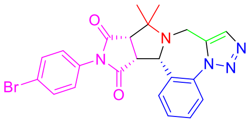

6c

White solid, 57% yield. M.P. 164–166 °C

^{1}H NMR (400 MHz, cdcl$_3$) δ 8.27 – 8.21 (m, 1H), 7.63 (d, *J* = 8.0 Hz, 1H), 7.50 (s, 1H), 7.47 – 7.41 (m, 4H), 6.92 – 6.88 (m, 2H), 5.01 (d, *J* = 6.3 Hz, 1H), 4.06 (d, *J* = 15.2 Hz, 1H), 3.68 – 3.58 (m, 2H), 3.05 (d, *J* = 7.9 Hz, 1H), 1.36 (s, 3H), 1.28 (s, 3H).

^{13}C NMR (101 MHz, cdcl$_3$) δ 174.45, 173.54, 136.57, 134.74, 132.12, 131.16, 130.45, 128.80, 127.63, 126.07, 124.33, 122.31, 68.17, 62.81, 53.08, 47.79, 37.94, 24.93, 20.61.

HRMS (ESI-TOF, *m/z*): [M+H]$^+$ calculated for $C_{23}H_{21}BrN_5O_2$ 478.0879, found 478.0883

(11aS,14aR,14bS)-13-benzyl-11,11-dimethyl-11,11a,14a,14b-tetrahydro-9H,12H-benzo[f]pyrrolo[3',4':3,4]pyrrolo[1,2-d][1,2,3]triazolo[1,5-a][1,4]diazepine-12,14(13H)-dione ***(6d)***

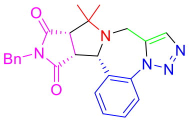

6d

White solid, 60% yield. M.P. 225–227°C

^{1}H NMR (400 MHz, cdcl$_3$) δ 8.28 – 8.23 (m, 1H), 7.57 (d, *J* = 8.4 Hz, 1H), 7.48 (d, *J* = 0.4 Hz, 1H), 7.44 (ddd, *J* = 12.2, 6.1, 3.8 Hz, 2H), 7.15 – 7.06 (m, 3H), 6.92 – 6.88 (m, 2H), 4.99 (d, *J* = 6.5 Hz, 1H), 4.49 (d, *J* = 14.5 Hz, 1H), 4.36 (d, *J* = 14.5 Hz, 1H), 3.99 (d, *J* = 15.2 Hz, 1H), 3.56 – 3.49 (m, 2H), 2.88 (d, *J* = 7.7 Hz, 1H), 1.20 (s, 3H), 1.13 (s, 3H).

^{13}C NMR (101 MHz, cdcl$_3$) δ 175.41, 174.58, 136.85, 135.15, 134.69, 131.28, 130.35, 128.71, 128.50, 127.63 – 127.22 (m), 126.35, 124.39, 68.31, 62.47, 52.85, 47.71, 42.02, 37.76, 24.43, 20.36, 14.19.

HRMS (ESI-TOF, *m/z*): [M+H]$^+$ calculated for $C_{24}H_{24}N_5O_2$ 414.1930, found 414.1932

(11aS,14aR,14bS)-11,11,13-trimethyl-11,11a,14a,14b-tetrahydro-9H,12H-benzo[f]pyrrolo[3',4':3,4]pyrrolo[1,2-d][1,2,3]triazolo[1,5-a][1,4]diazepine-12,14(13H)-dione ***(6e)***

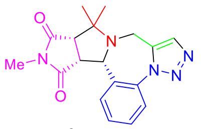

6e

White solid, 63% yield. M.P. 181–183°C

^{1}H NMR (400 MHz, cdcl$_3$) δ 8.25 – 8.21 (m, 1H), 7.59 (dd, *J* = 11.4, 4.7 Hz, 1H), 7.47 – 7.39 (m, 3H), 4.92 (d, *J* = 6.7 Hz, 1H), 4.01 (d, *J* = 15.2 Hz, 1H), 3.54 (d, *J* = 8.3 Hz, 1H), 3.52 – 3.49 (m, 1H), 2.90 (d, *J* = 7.9 Hz, 1H), 2.74 (s, 3H), 1.28 (s, 3H), 1.21 (s, 3H).

^{13}C NMR (101 MHz, cdcl$_3$) δ 175.88, 174.88, 136.52, 134.62, 131.41, 130.72, 128.68, 127.43, 126.25, 124.10, 62.51, 53.10, 47.64, 38.20, 24.90, 24.69, 20.75, 14.13.

HRMS (ESI-TOF, *m/z*): [M+H]$^+$ calculated for $C_{18}H_{20}N_5O_2$ 338.1617, found 338.1620

(11aS,14aR,14bS)-2-bromo-13-ethyl-11,11-dimethyl-11,11a,14a,14b-tetrahydro-9H,12H-benzo[f]pyrrolo[3',4':3,4]pyrrolo[1,2-d][1,2,3]triazolo[1,5-a][1,4]diazepine-12,14(13H)-dione ***(6f)***

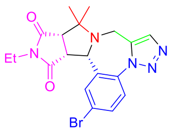

6f

White solid, 59% yield. M.P. 254–256°C

^{1}H NMR (400 MHz, cdcl$_3$) δ 8.10 (t, *J* = 9.9 Hz, 1H), 7.77 (s, 1H), 7.56 (dd, *J* = 8.9, 2.2 Hz, 1H), 7.46 (s, 1H), 4.86 (d, *J* = 6.5 Hz, 1H), 4.01 (d, *J* = 15.3 Hz, 1H), 3.54 (d, *J* = 15.3 Hz, 1H), 3.48 – 3.43 (m, 1H), 3.32 (qd, *J* = 7.1, 1.4 Hz, 2H), 2.87 (d, *J* = 7.7 Hz, 1H), 1.27 (d, *J* = 4.1 Hz, 3H), 1.21 (s, 3H), 0.89 (t, *J* = 7.2 Hz, 3H).

^{13}C NMR (101 MHz, cdcl$_3$) δ 175.30, 174.38, 136.60, 133.90, 131.79, 130.57, 128.42, 125.74, 121.20, 67.55, 62.45, 52.85, 47.46, 37.82, 33.76, 24.61, 20.62, 12.73.

HRMS (ESI-TOF, m/z): [M+H]$^+$ calculated for $C_{19}H_{21}BrN_5O_2$ 430.0879, found 430.0883

(11aS,14aR,14bS)-13-ethyl-11,11-dimethyl-4-(trifluoromethyl)-11,11a,14a,14b-tetrahydro-9H,12H-benzo[f]pyrrolo[3',4':3,4]pyrrolo[1,2-d][1,2,3]triazolo[1,5-a][1,4]diazepine-12,14(13H)-dione ***(6g)***

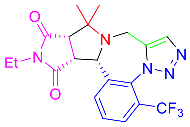

6g

White solid, 35% yield. M.P. 210–212°C

^{1}H NMR (400 MHz, cdcl$_3$) δ 9.11 (s, 1H), 7.92 (dd, *J* = 8.0, 1.0 Hz, 1H), 7.70 (t, *J* = 8.0 Hz, 1H), 7.61 (s, 1H), 4.03 (d, *J* = 16.1 Hz, 1H), 3.78 (d, *J* = 16.0 Hz, 1H), 3.63 – 3.43 (m, 4H), 2.83 (d, *J* = 7.5 Hz, 1H), 1.26 (s, 3H), 1.15 (s, 6H).

^{13}C NMR (101 MHz, cdcl$_3$) δ 175.84, 175.25, 135.08, 131.44, 130.98, 129.37, 128.52, 124.20, 62.24, 53.69, 50.80, 46.73, 36.12, 34.02, 31.55, 24.80, 24.13, 22.61, 14.08, 12.93.

^{19}F NMR (376 MHz, cdcl$_3$) δ -58.15.

HRMS (ESI-TOF, m/z): [M+H]$^+$ calculated for $C_{20}H_{21}F_3N_5O_2$ 420.1647, found 420.1646

(11S,11aS,14aR,14bS)-13-ethyl-11-isopropyl-11,11a,14a,14b-tetrahydro-9H,12H-benzo[f]pyrrolo[3',4':3,4]pyrrolo[1,2-d][1,2,3]triazolo[1,5-a][1,4]diazepine-12,14(13H)-dione ***(6h)***

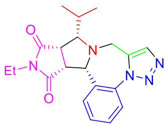

6h

White solid, 52% yield. M.P. 90–92°C

^{1}H NMR (400 MHz, cdcl$_3$) δ 7.86 (d, *J* = 7.8 Hz, 1H), 7.61 – 7.51 (m, 2H), 7.48 (dd, *J* = 5.1, 1.0 Hz, 2H), 5.04 (d, *J* = 9.8 Hz, 1H), 3.98 (d, *J* = 16.1 Hz, 1H), 3.79 (d, *J* = 16.1 Hz, 1H), 3.34 (dd, *J* = 6.2, 2.5 Hz, 1H), 3.32 – 3.25 (m, 1H), 3.20 – 3.07 (m, 2H), 2.97 (dd, *J* = 8.9, 2.5 Hz, 1H), 1.83 (dt, *J* = 13.3, 6.6 Hz, 1H), 1.04 (dd, *J* = 7.5, 6.8 Hz, 6H), 0.86 (t, *J* = 7.2 Hz, 3H).

^{13}C NMR (101 MHz, cdcl$_3$) δ 177.64, 174.69, 136.52, 135.53, 132.11, 131.25, 130.50, 129.20, 127.83, 124.17, 74.06, 69.75, 53.21, 48.97, 44.69, 34.36, 19.10, 18.65, 12.71.

HRMS (ESI-TOF, m/z): [M+H]$^+$ calculated for $C_{20}H_{24}N_5O_2$ 366.1930, found 366.1932

(11S,11aS,14aR,14bS)-2-bromo-11-isopropyl-13-methyl-11,11a,14a,14b-tetrahydro-9H,12H-benzo[f]pyrrolo[3',4':3,4]pyrrolo[1,2-d][1,2,3]triazolo[1,5-a][1,4]diazepine-12,14(13H)-dione ***(6i)***

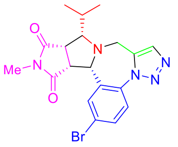

6i

White solid, 44% yield. M.P. 200–201°C

^{1}H NMR (400 MHz, cdcl$_3$) δ 7.74 (d, *J* = 8.5 Hz, 1H), 7.70 (dd, *J* = 8.5, 2.0 Hz, 1H), 7.65 (d, *J* = 2.0 Hz, 1H), 7.56 (d, *J* = 0.5 Hz, 1H), 4.94 (d, *J* = 9.7 Hz, 1H), 3.96 (d, *J* = 16.1 Hz, 1H), 3.79 (d, *J* = 16.2 Hz, 1H), 3.36 (dd, *J* = 6.3, 2.4 Hz, 1H), 3.28 (t, *J* = 9.3 Hz, 1H), 2.97 (dd, *J* = 8.9, 2.4 Hz, 1H), 2.60 (s, 3H), 1.81 (dt, *J* = 13.2, 6.6 Hz, 1H), 1.03 (dd, *J* = 11.0, 6.7 Hz, 6H).

^{13}C NMR (101 MHz, cdcl$_3$) δ 177.81, 174.72, 135.54, 134.92, 133.60, 131.37, 129.69, 125.65, 122.90, 74.60, 69.21, 53.40, 49.04, 44.90, 34.52, 25.50, 19.23, 18.55.

HRMS (ESI-TOF, m/z): [M+H]$^+$ calculated for $C_{20}H_{24}N_5O_2$ 430.0879, found 430.0882

(11S,11aS,14aR,14bS)-2-chloro-13-ethyl-11-isopropyl-11,11a,14a,14b-tetrahydro-9H,12H-benzo[f]pyrrolo[3',4':3,4]pyrrolo[1,2-d][1,2,3]triazolo[1,5-a][1,4]diazepine-12,14(13H)-dione ***(6j)***

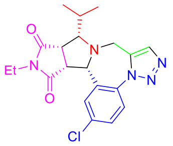

6j

White solid, 47% yield. M.P. 78–80°C

^{1}H NMR (400 MHz, cdcl$_3$) δ 7.81 (d, *J* = 8.5 Hz, 1H), 7.58 – 7.44 (m, 3H), 4.96 (d, *J* = 9.7 Hz, 1H), 3.96 (d, *J* = 16.1 Hz, 1H), 3.82 – 3.77 (m, 1H), 3.34 – 3.27 (m, 2H), 3.13 (dt, *J* = 14.0, 6.7 Hz, 2H), 2.98 (dd, *J* = 8.9, 2.5 Hz, 1H), 1.83 (td, *J* = 13.2, 6.6 Hz, 1H), 1.03 (dd, *J* = 8.7, 6.7 Hz, 6H), 0.86 (t, *J* = 7.2 Hz, 3H).

^{13}C NMR (101 MHz, cdcl$_3$) δ 177.45, 174.50, 135.51, 135.05, 134.81, 131.98, 131.34, 130.48, 129.51, 125.43, 74.12, 69.22, 53.10, 48.89, 44.57, 34.46, 34.19, 19.06, 18.62, 12.71.

HRMS (ESI-TOF, m/z): [M+H]$^+$ calculated for $C_{20}H_{23}ClN_5O$ 400.1540, found 400.1542

(11R,11aS,14aR,14bS)-13-ethyl-11-phenyl-11,11a,14a,14b-tetrahydro-9H,12H-benzo[f]pyrrolo[3',4':3,4]pyrrolo[1,2-d][1,2,3]triazolo[1,5-a][1,4]diazepine-12,14(13H)-dione ***(6k)***

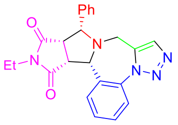

6k

White solid, 55% yield. M.P. 148–150°C

^{1}H NMR (400 MHz, cdcl$_3$) δ 7.93 (ddd, *J* = 6.6, 4.1, 1.7 Hz, 2H), 7.65 (dtt, *J* = 6.8, 4.4, 2.3 Hz, 2H), 7.60 (t, *J* = 2.8 Hz, 1H), 7.50 (dt, *J* = 5.3, 3.0 Hz, 2H), 7.43 (ddd, *J* = 7.4, 5.7, 2.8 Hz, 2H), 7.38 – 7.32 (m, 1H), 3.89 (dd, *J* = 12.1, 8.2 Hz, 2H), 3.79 (d, *J* = 15.9 Hz, 1H), 3.64 (d, *J* = 15.8 Hz, 1H), 3.47 (qd, *J* = 7.2, 2.8 Hz, 2H), 3.34 (dd, *J* = 9.3, 7.1 Hz, 2H), 1.10 (t, *J* = 7.2 Hz, 3H).

^{13}C NMR (101 MHz, cdcl$_3$) δ 176.04, 174.89, 139.09, 136.18, 132.70, 131.98, 130.05, 129.75, 129.23, 128.64, 127.94 – 127.51 (m), 123.46, 68.88, 64.82, 52.45, 44.79, 39.12, 33.83, 12.98.

HRMS (ESI-TOF, m/z): [M+H]$^+$ calculated for $C_{23}H_{22}N_5O_2$ 400.1773, found 400.1774

(11R,11aS,14aR,14bS)-2-bromo-13-ethyl-11-phenyl-11,11a,14a,14b-tetrahydro-9H,12H-benzo[f]pyrrolo[3',4':3,4]pyrrolo[1,2-d][1,2,3]triazolo[1,5-a][1,4]diazepine-12,14(13H)-dione ***(6l)***

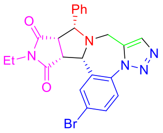

6l

White solid, 52% yield. M.P. 190–192°C

Mixture, dr 2:1

^{1}H NMR (400 MHz, cdcl$_3$) δ 8.28 (d, *J* = 1.7 Hz, 1H), 8.11 (d, *J* = 8.8 Hz, 1H), 7.73 – 7.62 (m, 2H), 7.43 – 7.35 (m, 5H), 4.26 (d, *J* = 5.5 Hz, 1H), 4.07 (d, *J* = 9.3 Hz, 1H), 3.94 – 3.89 (m, 1H), 3.60 (dd, *J* = 5.0, 2.7 Hz, 1H), 3.44 – 3.38 (m, 2H), 3.32 (dd, *J* = 7.1, 4.9 Hz, 2H), 0.97 (t, *J* = 7.2 Hz, 3H).

^{13}C NMR (101 MHz, cdcl$_3$) δ 174.71, 173.98, 135.23, 134.28, 133.90, 132.25, 131.75, 129.11 – 129.06 (m), 128.81, 128.04, 125.40, 121.98, 69.78, 69.29, 48.29, 47.53, 41.32, 33.93, 12.87.

HRMS (ESI-TOF, m/z): $[M+H]^+$ calculated for $C_{23}H_{21}BrN_5O_2$ 478.0879, found 478.0883

3. NMR Spectra of Products

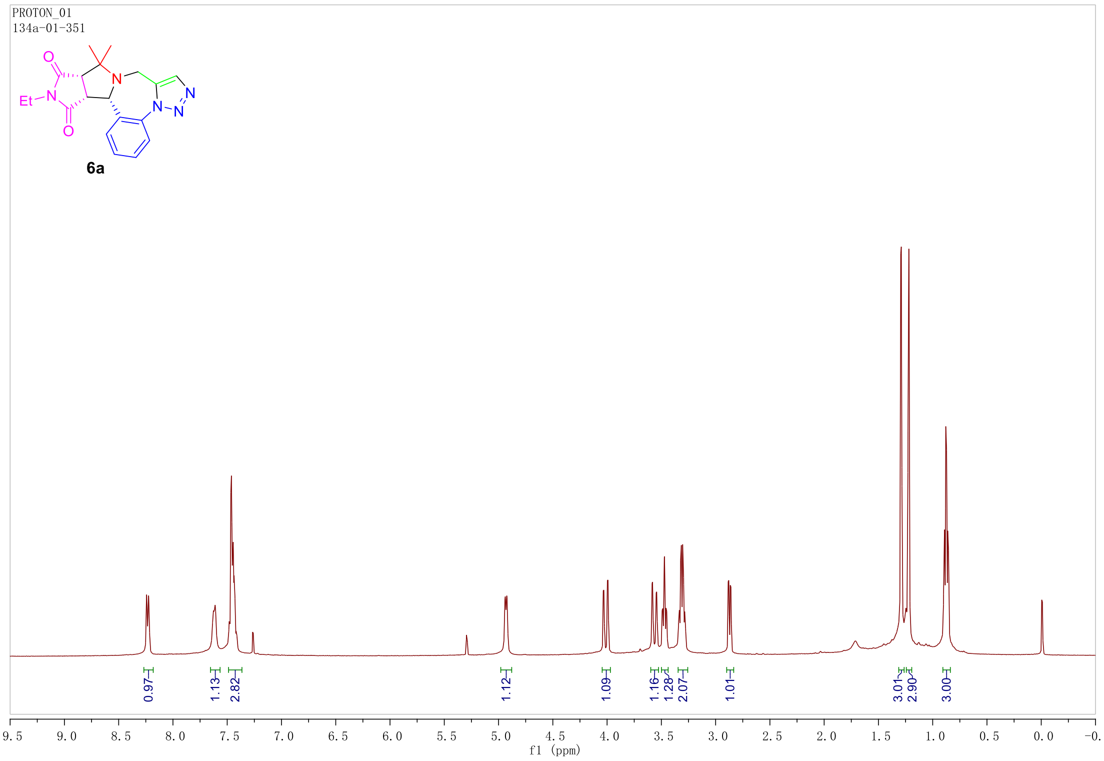

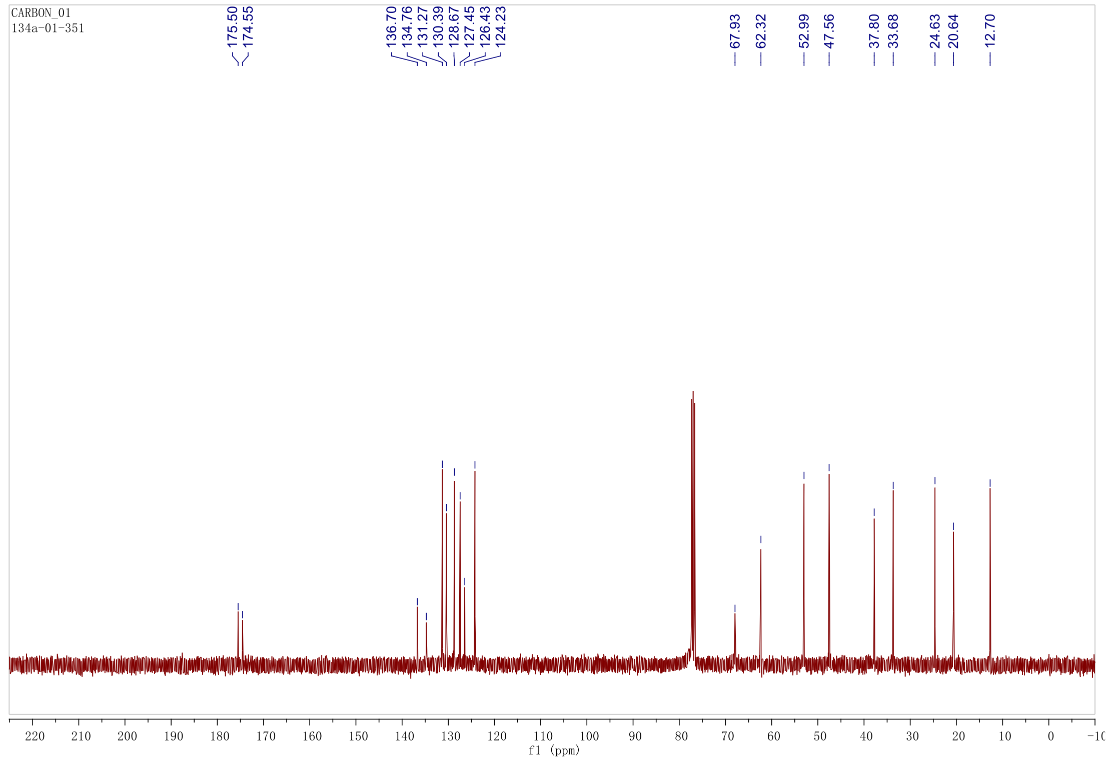

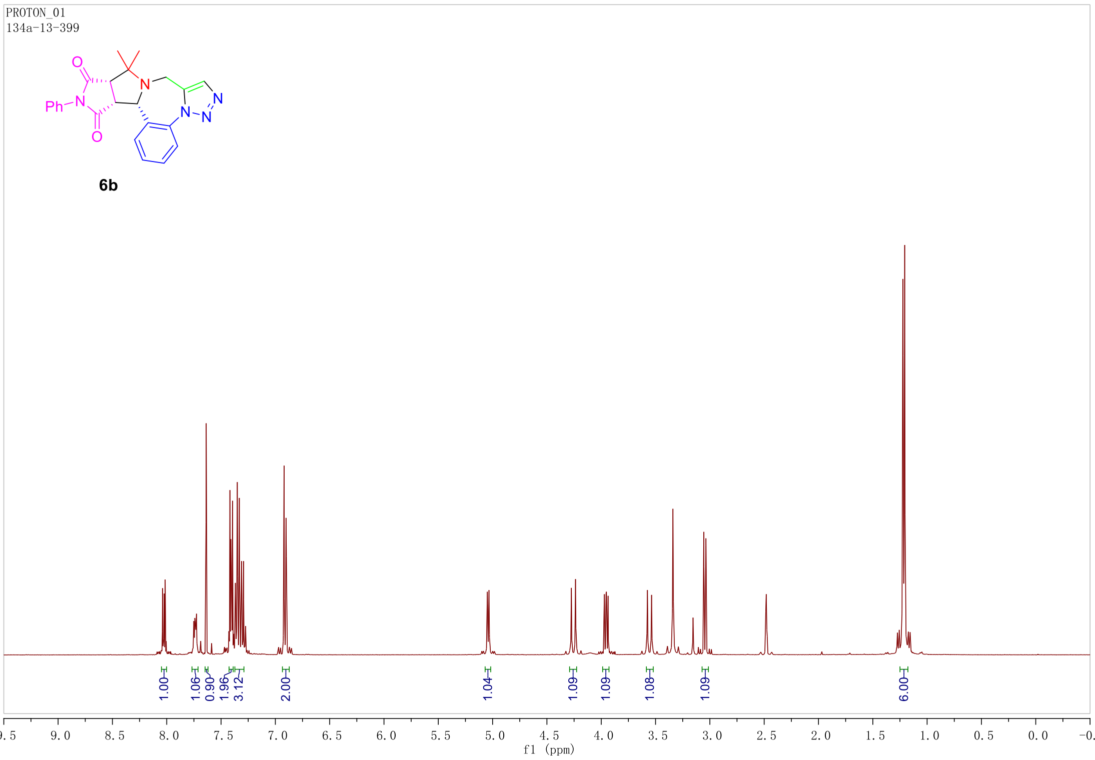

PROTON_01
134a-13-399
O
Ph
N
O
N
N
N
N
1.00
1.06
0.90
1.96
3.12
2.00
1.04
1.09
1.09
1.08
1.09
6.00
f1 (ppm)

6b

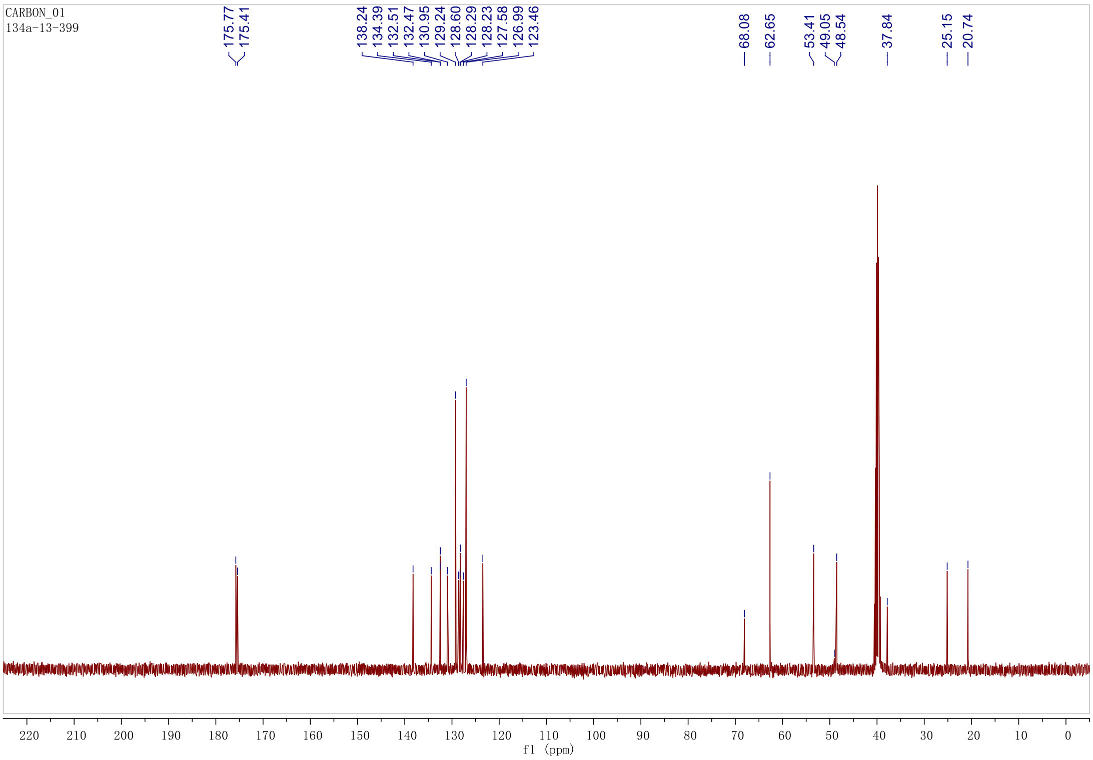

CARBON_01
134a-13-399
175.77
175.41
138.24
134.39
132.51
132.47
130.95
129.24
128.60
128.29
128.23
127.58
126.99
123.46
68.08
62.65
53.41
49.05
48.54
37.84
25.15
20.74
f1 (ppm)

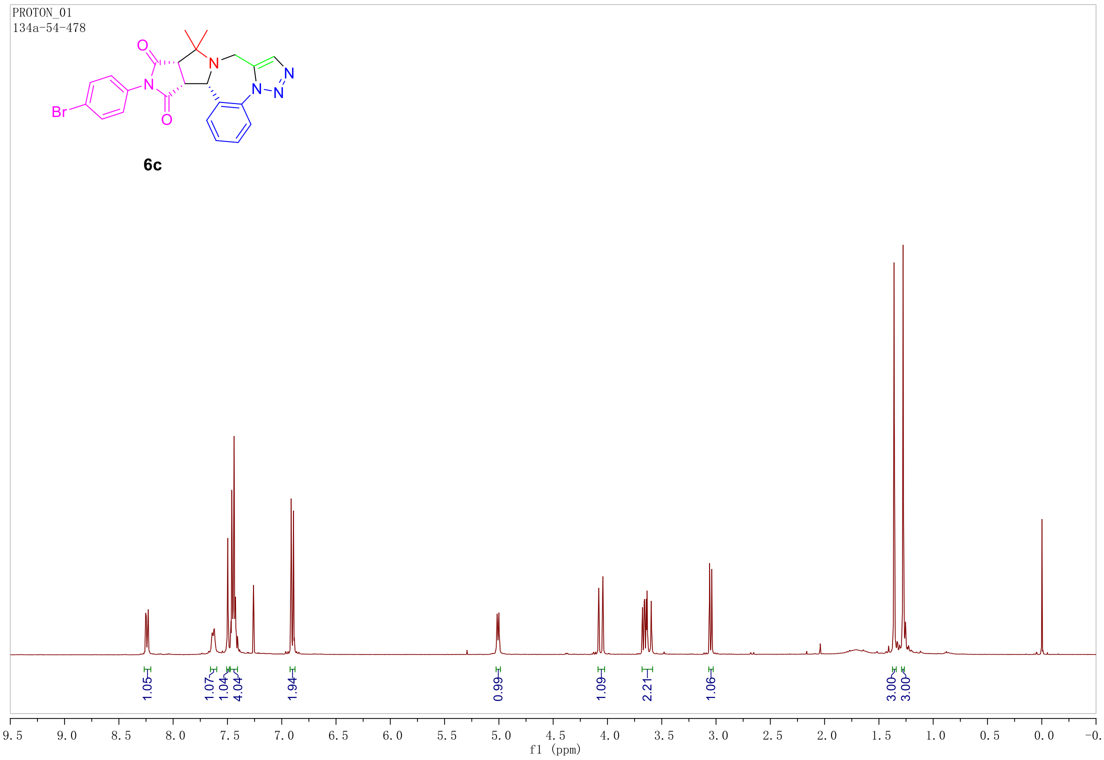
PROTON_01
134a-54-478
6c
1.05
1.07
1.04
4.04
1.94
0.99
1.09
2.21
1.06
3.00
3.00
f1 (ppm)

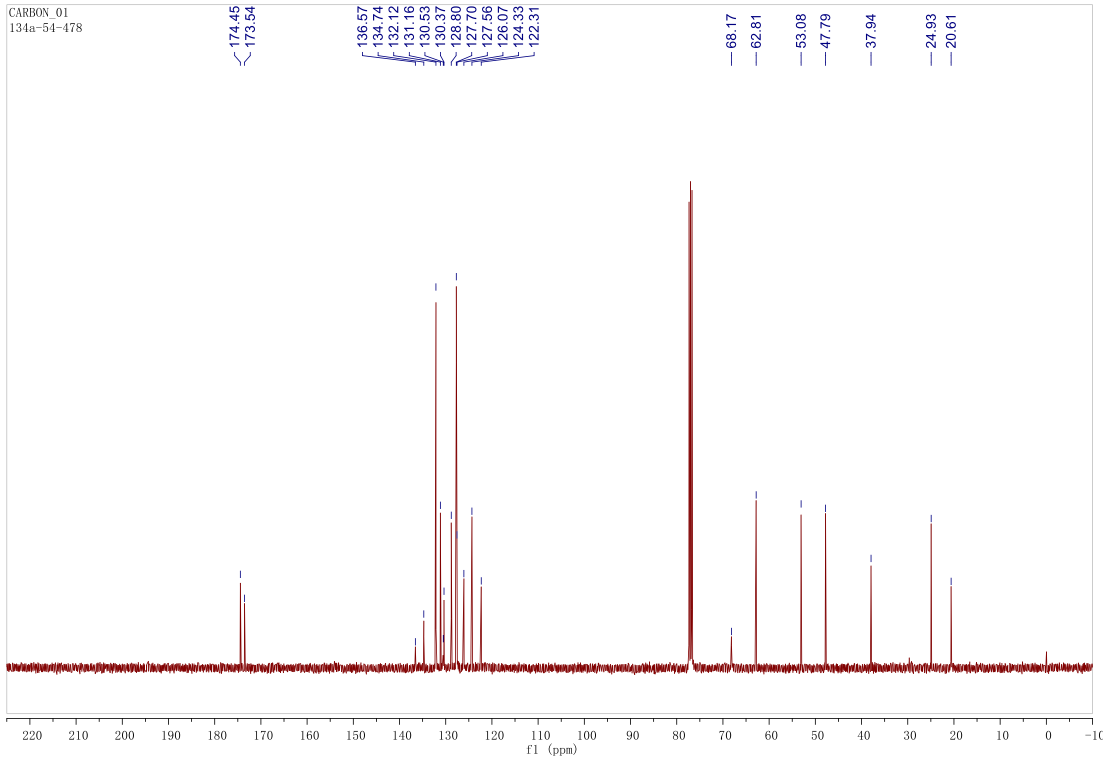
CARBON_01
134a-54-478
174.45
173.54
136.57
134.74
132.12
131.16
130.53
130.37
128.80
127.70
127.56
126.07
124.33
122.31
68.17
62.81
53.08
47.79
37.94
24.93
20.61
f1 (ppm)

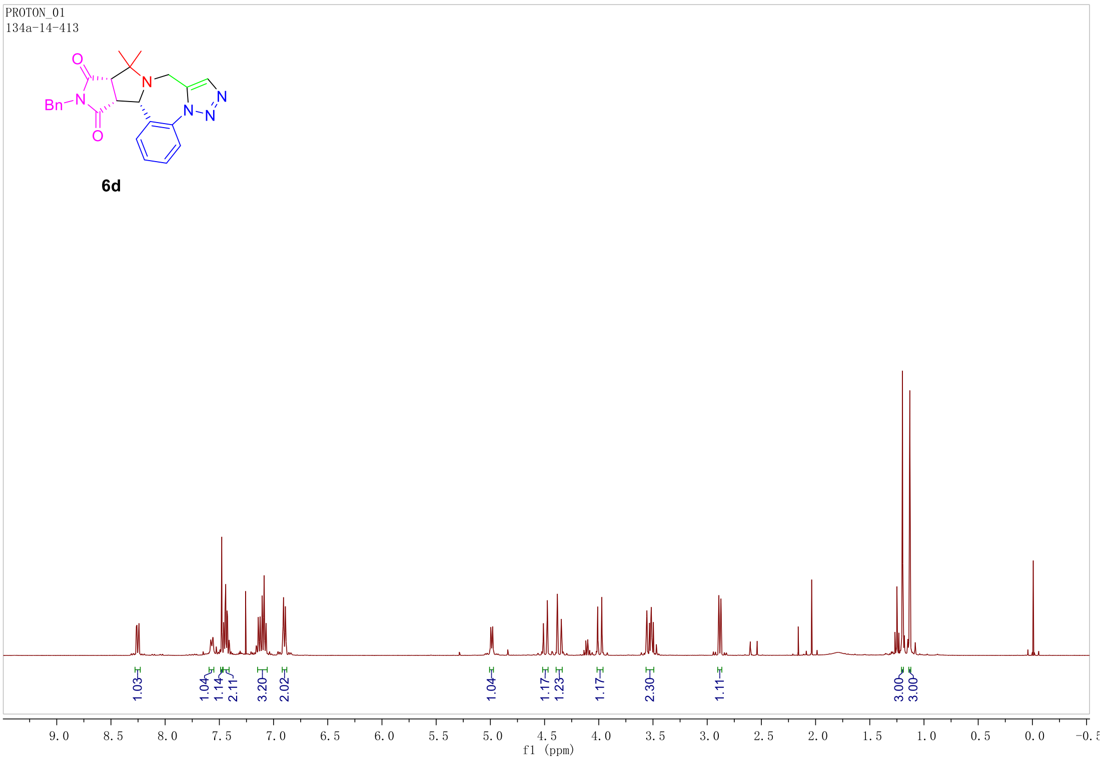
PROTON_01
134a-14-413
Bn
N
O
O
N
N
N
N
6d
1.03
1.04
1.14
2.11
3.20
2.02
1.04
1.17
1.23
1.17
2.30
1.11
3.00
3.00
9.0 8.5 8.0 7.5 7.0 6.5 6.0 5.5 5.0 4.5 4.0 3.5 3.0 2.5 2.0 1.5 1.0 0.5 0.0
f1 (ppm)

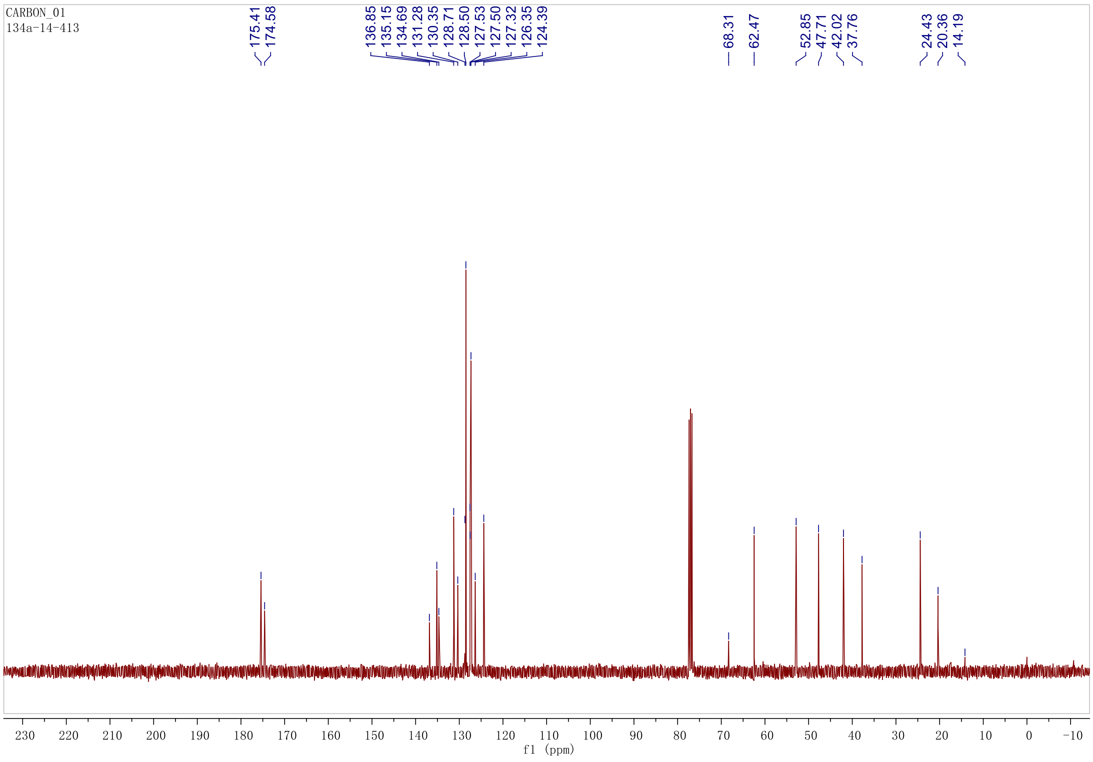
CARBON_01
134a-14-413
175.41
174.58
136.85
135.15
134.69
131.28
130.35
128.71
128.50
127.53
127.50
127.32
126.35
124.39
68.31
62.47
52.85
47.71
42.02
37.76
24.43
20.36
14.19
230 220 210 200 190 180 170 160 150 140 130 120 110 100 90 80 70 60 50 40 30 20 10 0 -10
f1 (ppm)

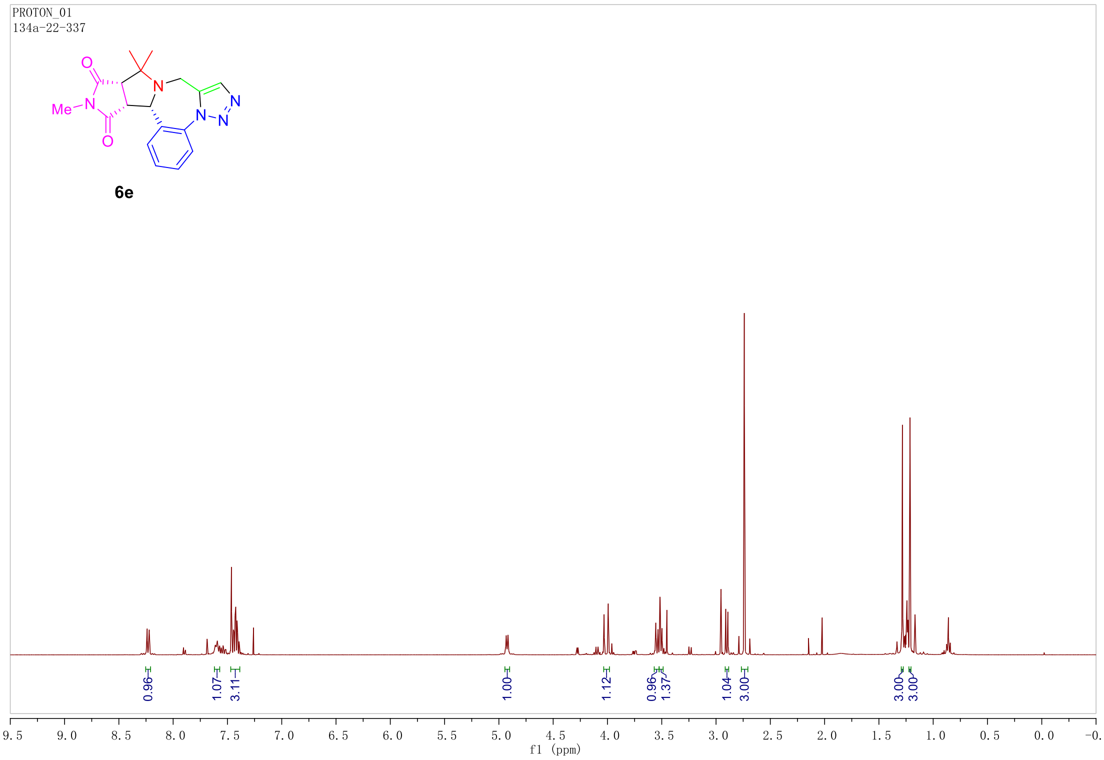
PROTON_01
134a-22-337
6e
0.96
1.07
3.11
1.00
1.12
0.96
1.37
1.04
3.00
3.00
3.00
9.5 9.0 8.5 8.0 7.5 7.0 6.5 6.0 5.5 5.0 4.5 4.0 3.5 3.0 2.5 2.0 1.5 1.0 0.5 0.0
f1 (ppm)

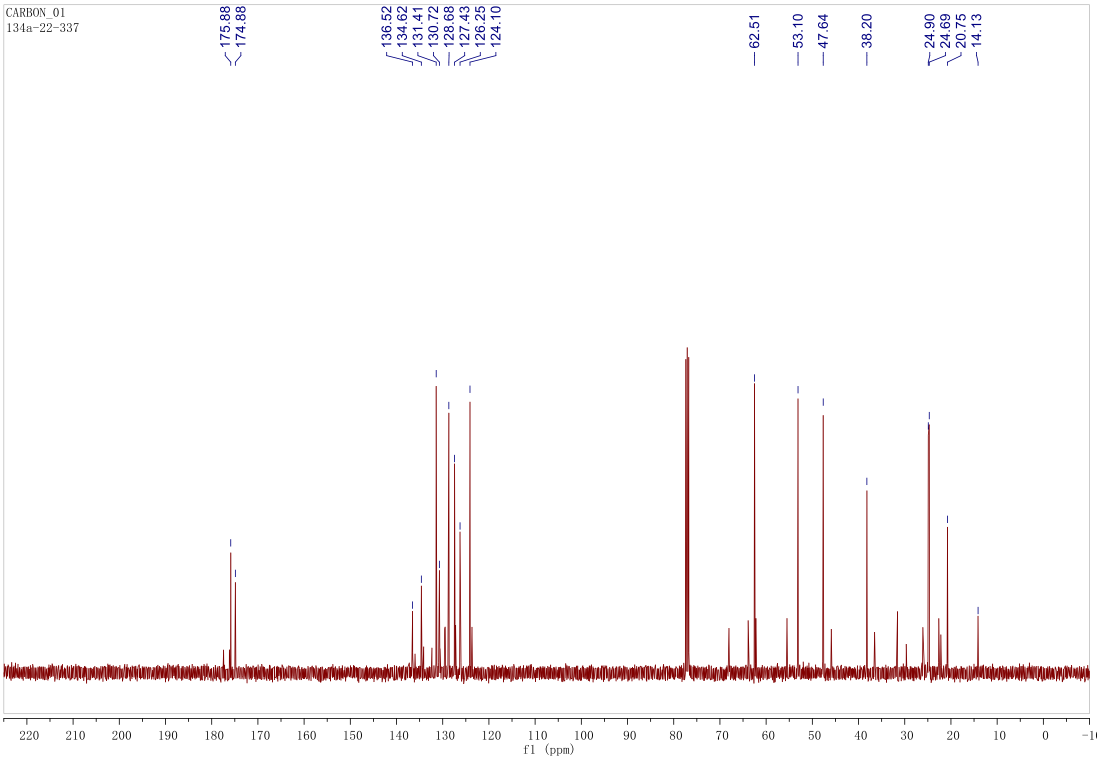
CARBON_01
134a-22-337
175.88
174.88
136.52
134.62
131.41
130.72
128.68
127.43
126.25
124.10
62.51
53.10
47.64
38.20
24.90
24.69
20.75
14.13
220 210 200 190 180 170 160 150 140 130 120 110 100 90 80 70 60 50 40 30 20 10 0
f1 (ppm)

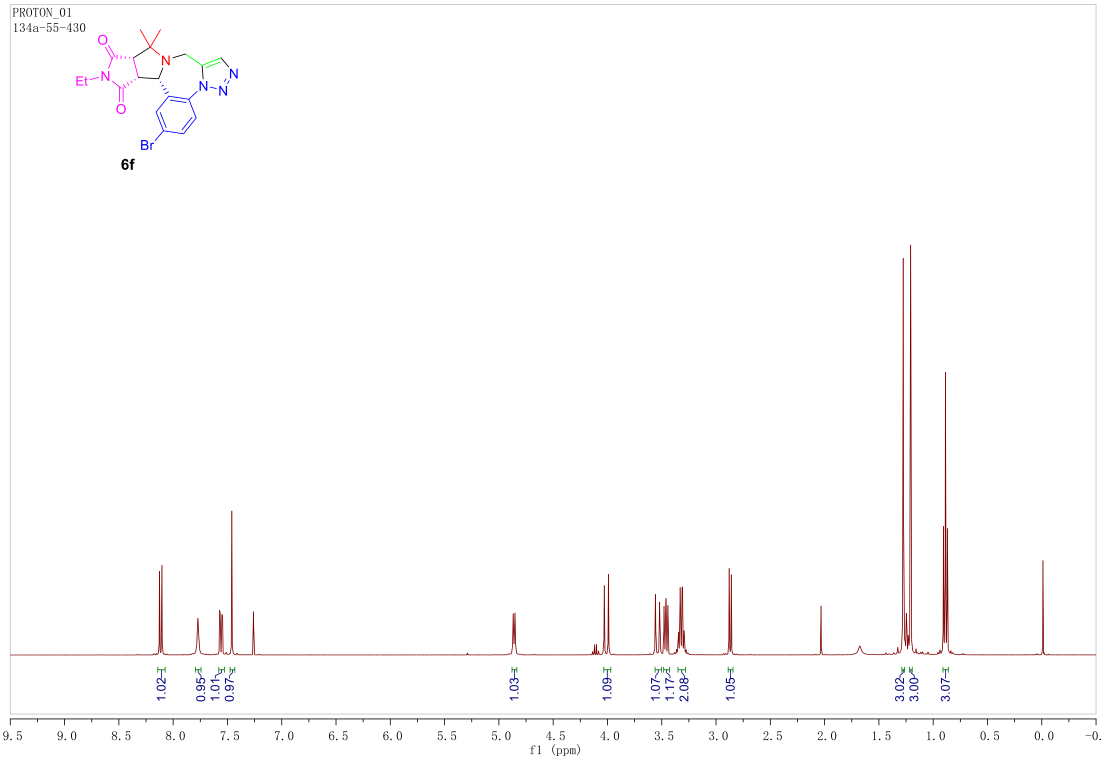

PROTON_01
134a-55-430
Et
N
O
O
N
N
N
N
Br
6f
1.02
0.95
1.01
0.97
1.03
1.09
1.07
1.17
2.08
1.05
3.02
3.00
3.07
9.5 9.0 8.5 8.0 7.5 7.0 6.5 6.0 5.5 5.0 4.5 4.0 3.5 3.0 2.5 2.0 1.5 1.0 0.5 0.0 -0.
f1 (ppm)

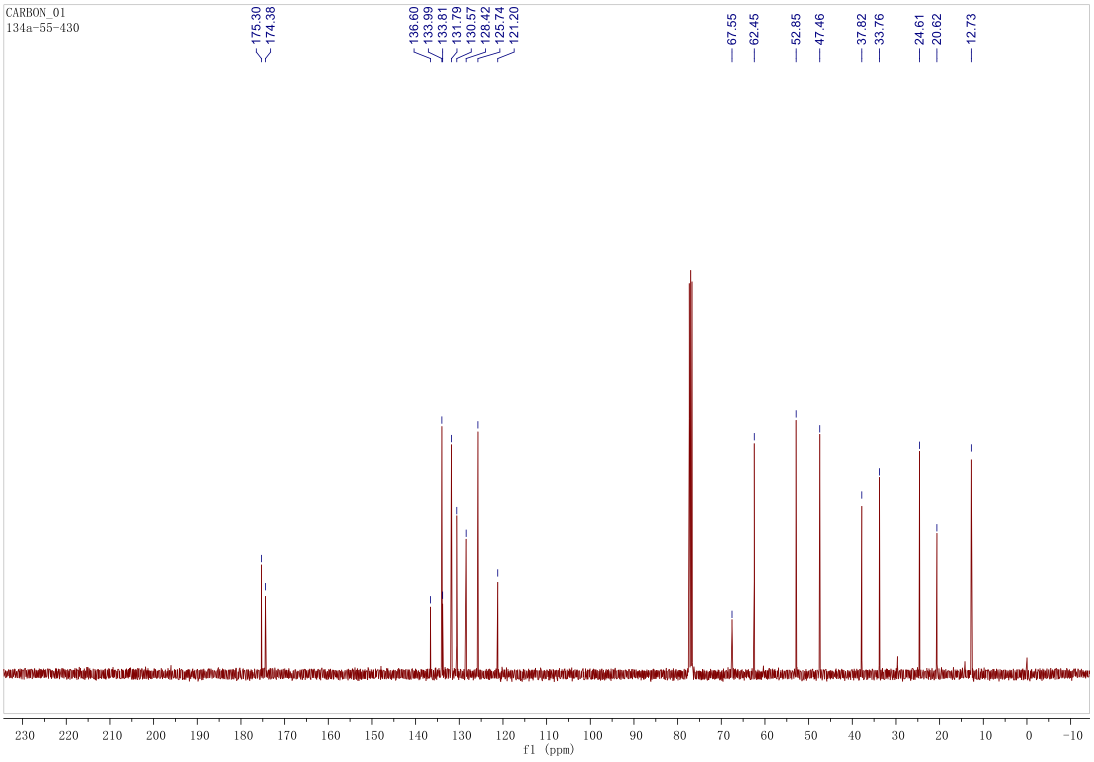

CARBON_01
134a-55-430
175.30
174.38
136.60
133.99
133.81
131.79
130.57
128.42
125.74
121.20
67.55
62.45
52.85
47.46
37.82
33.76
24.61
20.62
12.73
230 220 210 200 190 180 170 160 150 140 130 120 110 100 90 80 70 60 50 40 30 20 10 0 -10
f1 (ppm)

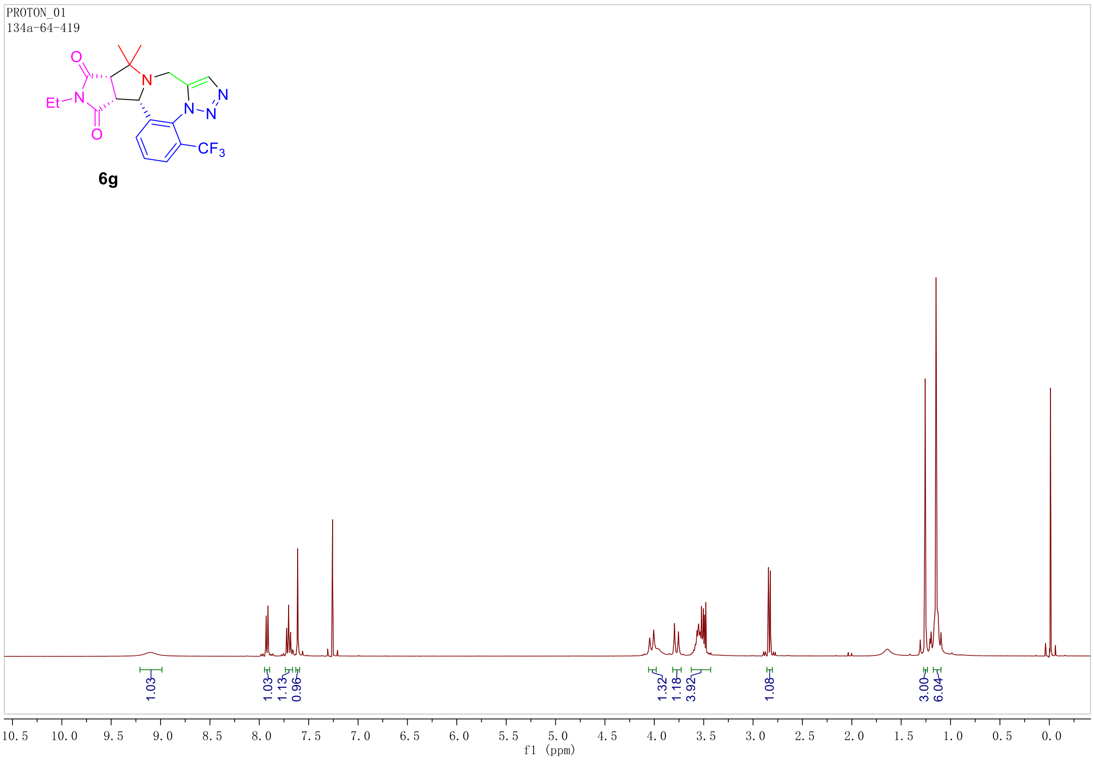
PROTON_01
134a-64-419
Et
N
O
O
N
N
N
N
CF3
6g
1.03
1.03
1.13
0.96
1.32
1.18
3.92
1.08
3.00
6.04
f1 (ppm)

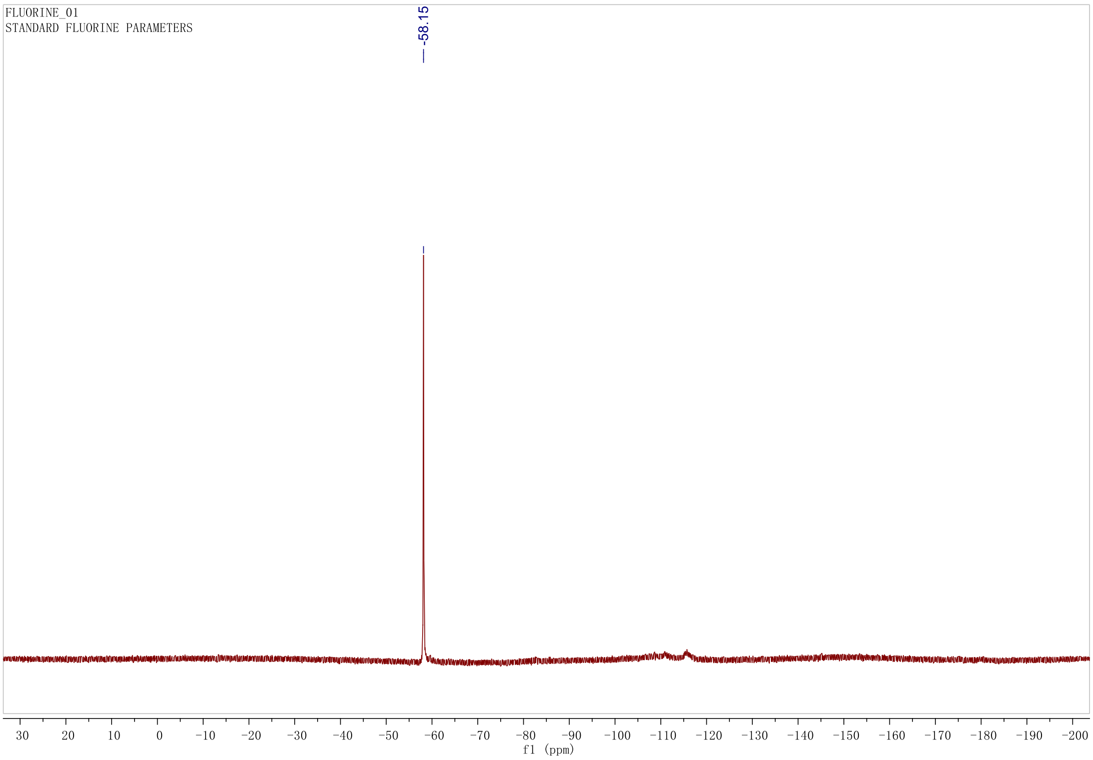
FLUORINE_01
STANDARD FLUORINE PARAMETERS
-58.15
f1 (ppm)

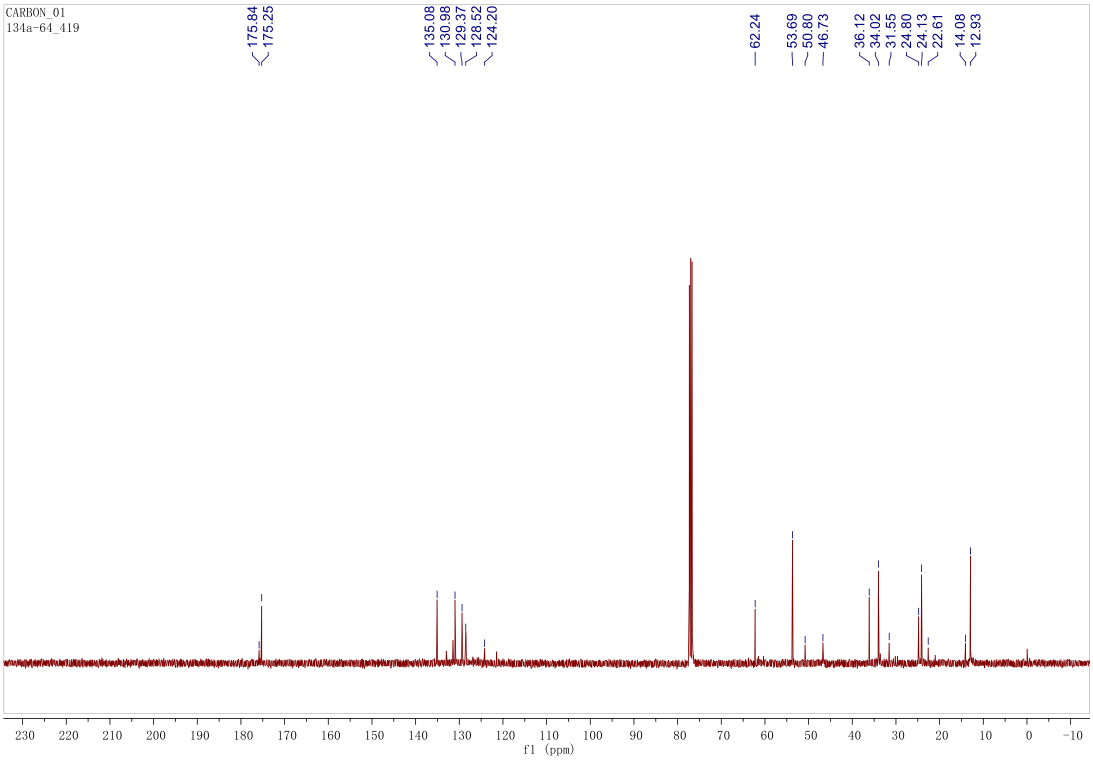

CARBON_01
134a-64_419
175.84
175.25
135.08
130.98
129.37
128.52
124.20
62.24
53.69
50.80
46.73
36.12
34.02
31.55
24.80
24.13
22.61
14.08
12.93
230
220
210
200
190
180
170
160
150
140
130
120
110
100
90
80
70
60
50
40
30
20
10
0
-10
f1 (ppm)

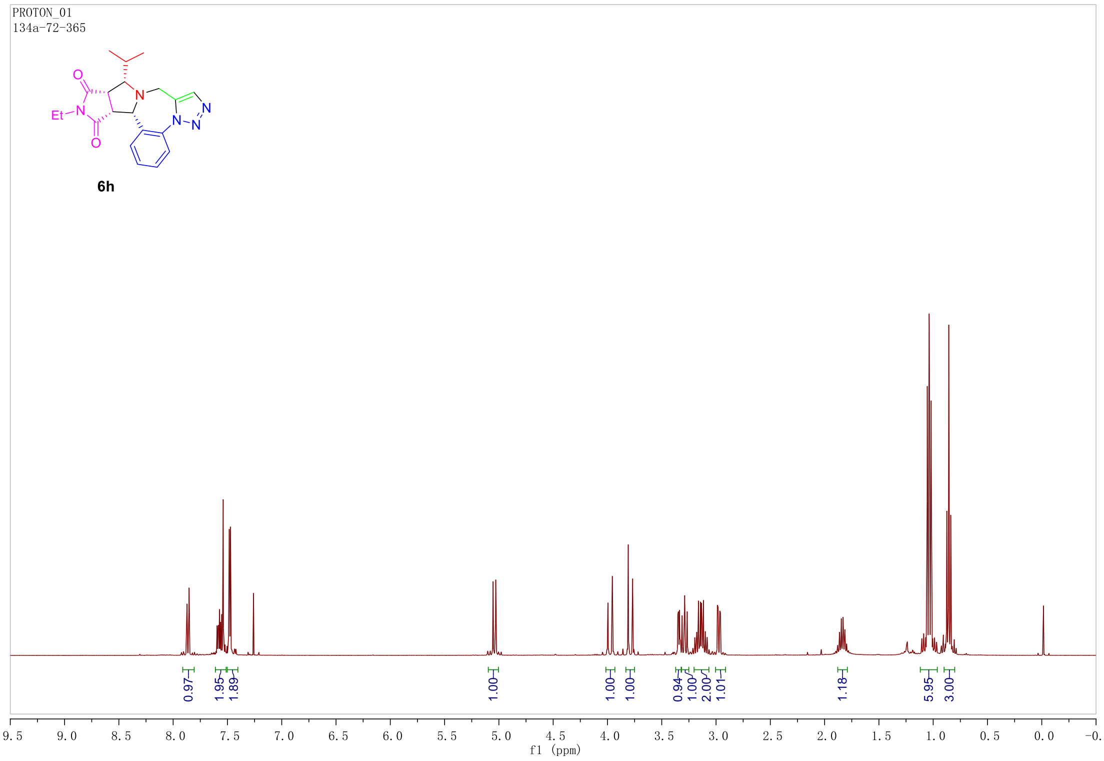
PROTON_01
134a-72-365
6h
0.97
1.95
1.89
1.00
1.00
1.00
0.94
1.00
2.00
1.01
1.18
5.95
3.00
f1 (ppm)

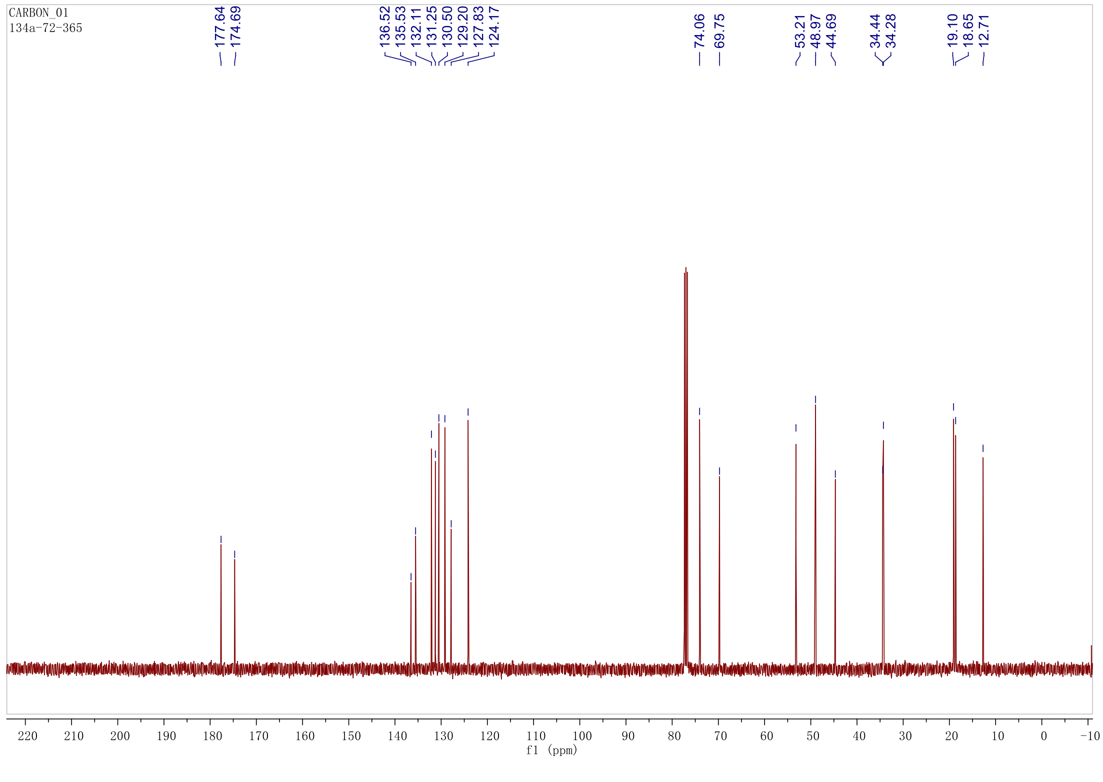
CARBON_01
134a-72-365
177.64
174.69
136.52
135.53
132.11
131.25
130.50
129.20
127.83
124.17
74.06
69.75
53.21
48.97
44.69
34.44
34.28
19.10
18.65
12.71
f1 (ppm)

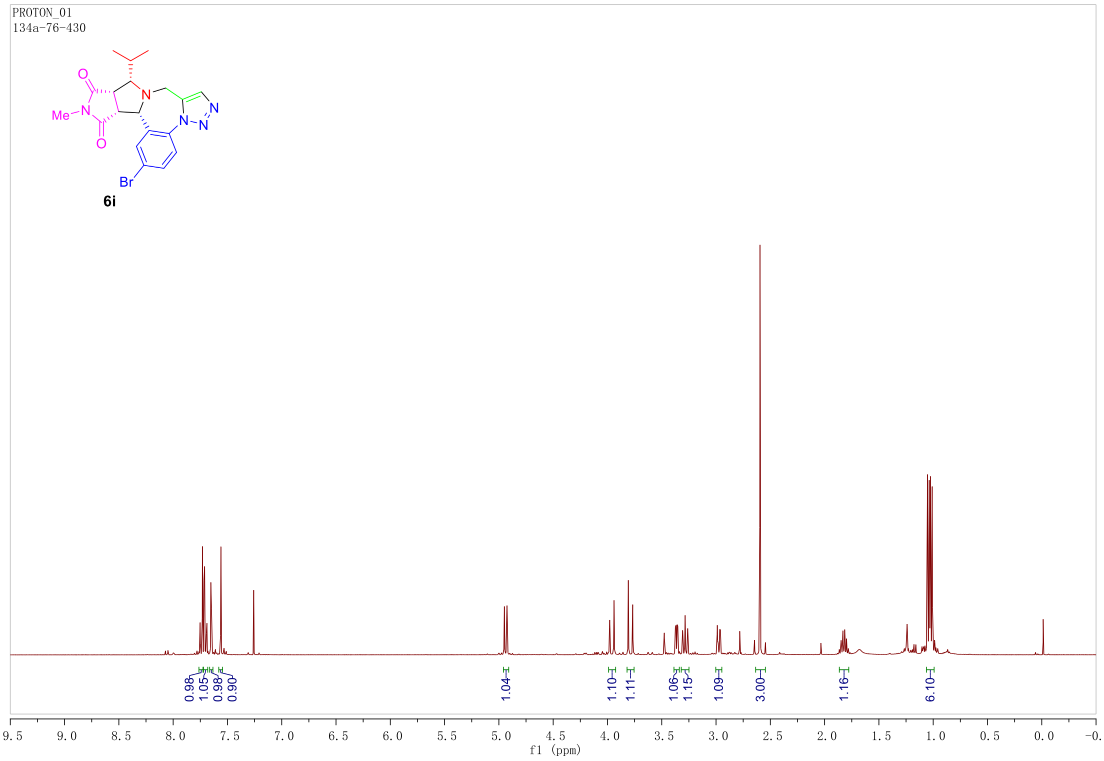
PROTON_01
134a-76-430
O
Me
N
O
N
N
N
N
Br
6i
0.98
1.05
0.98
0.90
1.04
1.10
1.11
1.06
1.15
1.09
3.00
1.16
6.10
9.5 9.0 8.5 8.0 7.5 7.0 6.5 6.0 5.5 5.0 4.5 4.0 3.5 3.0 2.5 2.0 1.5 1.0 0.5 0.0
f1 (ppm)

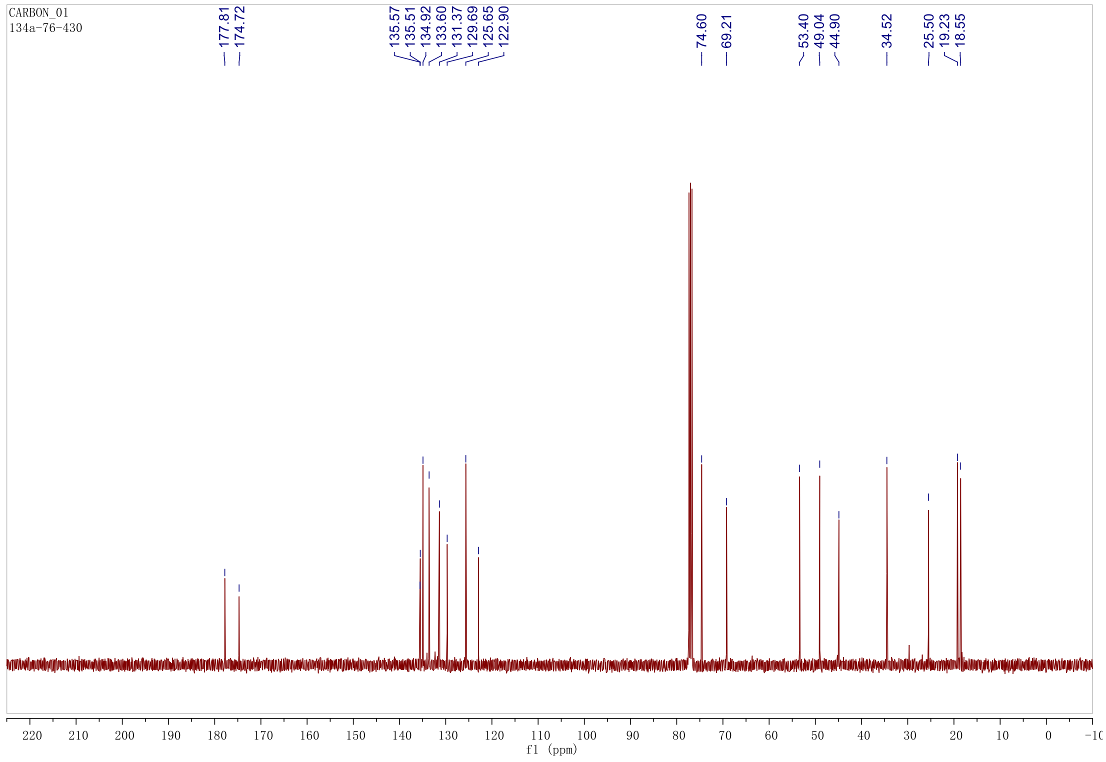
CARBON_01
134a-76-430
177.81
174.72
135.57
135.51
134.92
133.60
131.37
129.69
125.65
122.90
74.60
69.21
53.40
49.04
44.90
34.52
25.50
19.23
18.55
220 210 200 190 180 170 160 150 140 130 120 110 100 90 80 70 60 50 40 30 20 10 0
f1 (ppm)

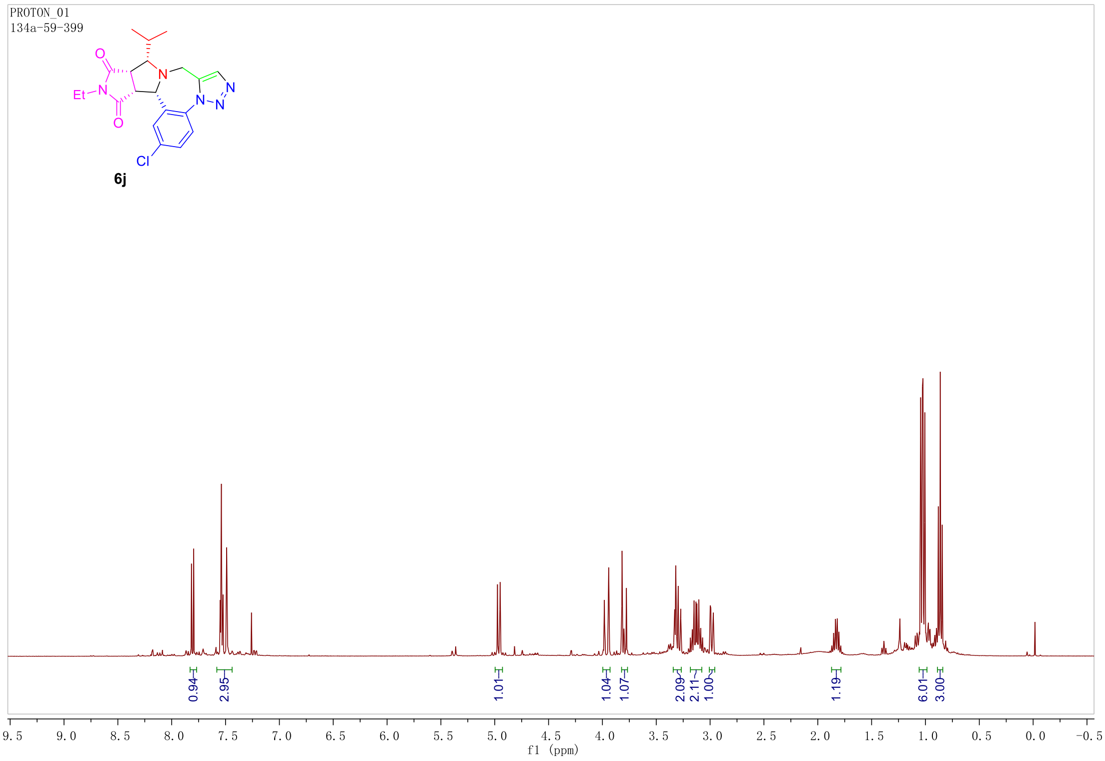
PROTON_01
134a-59-399
O
Et
N
O
N
N
N
N
Cl
6j
0.94
2.95
1.01
1.04
1.07
2.09
2.11
1.00
1.19
6.01
3.00
9.5 9.0 8.5 8.0 7.5 7.0 6.5 6.0 5.5 5.0 4.5 4.0 3.5 3.0 2.5 2.0 1.5 1.0 0.5 0.0 -0.5
f1 (ppm)

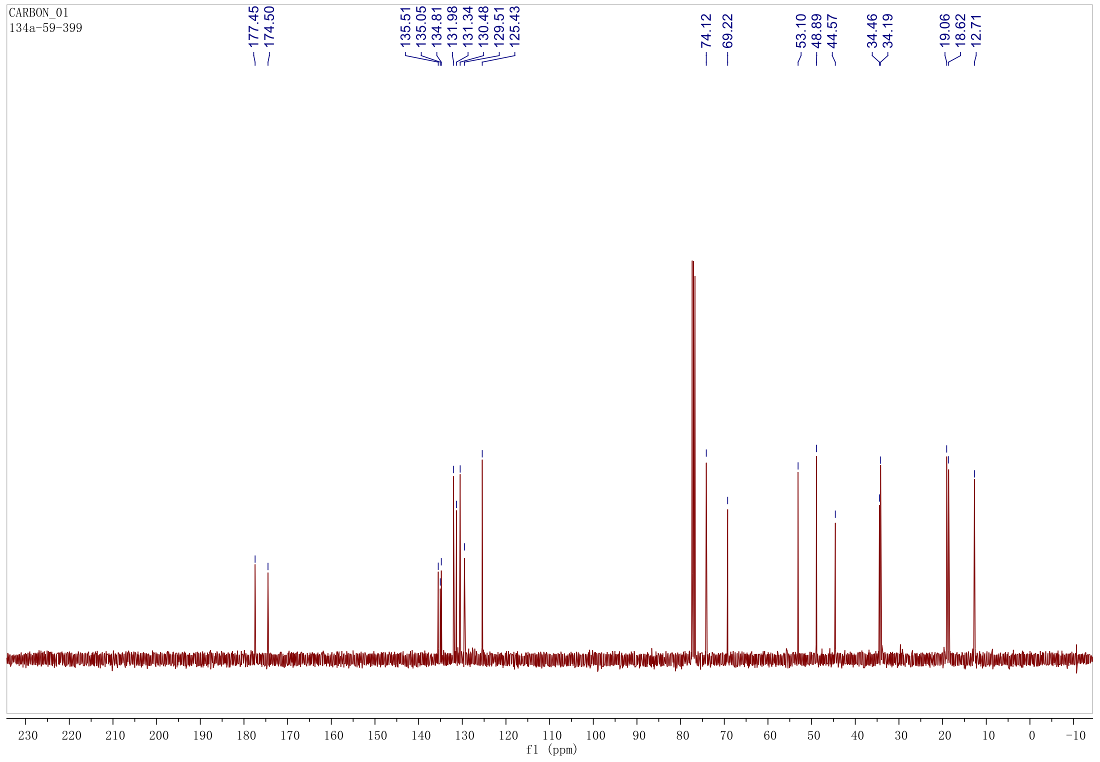
CARBON_01
134a-59-399
177.45
174.50
135.51
135.05
134.81
131.98
131.34
130.48
129.51
125.43
74.12
69.22
53.10
48.89
44.57
34.46
34.19
19.06
18.62
12.71
230 220 210 200 190 180 170 160 150 140 130 120 110 100 90 80 70 60 50 40 30 20 10 0 -10
f1 (ppm)

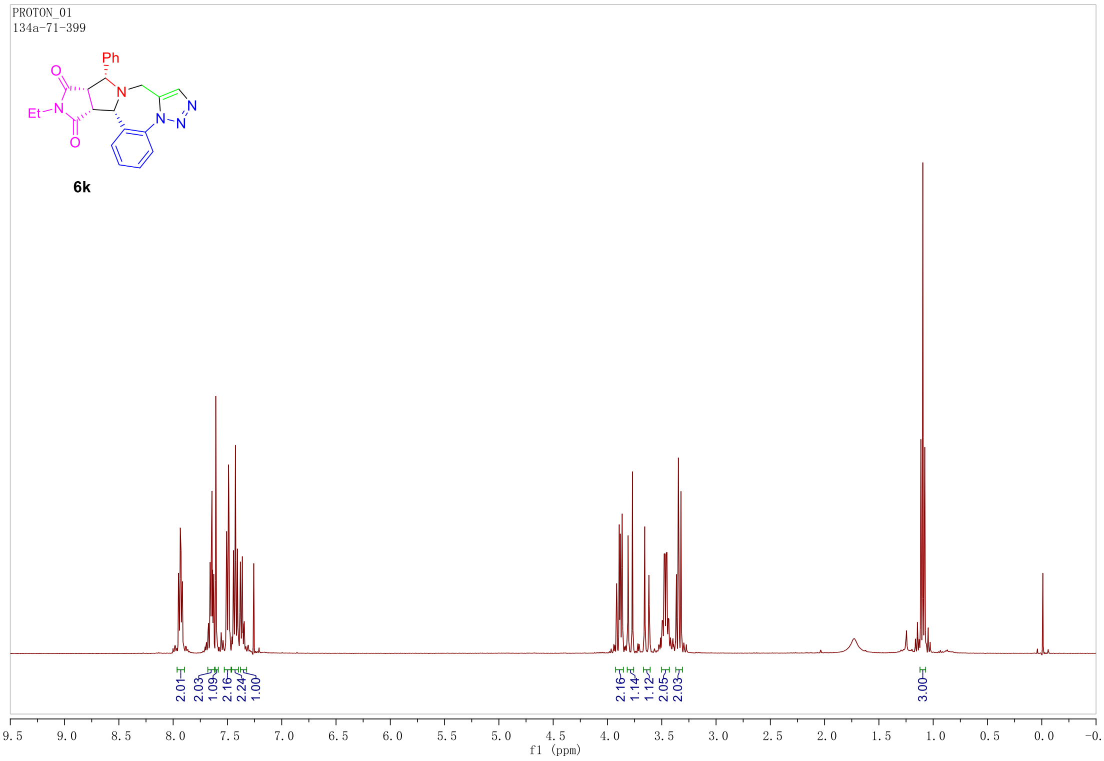
PROTON_01
134a-71-399
Ph
O
Et
N
N
N
N
N
O
6k
2.01
2.03
1.09
2.16
2.24
1.00
2.16
1.14
1.12
2.05
2.03
3.00
f1 (ppm)

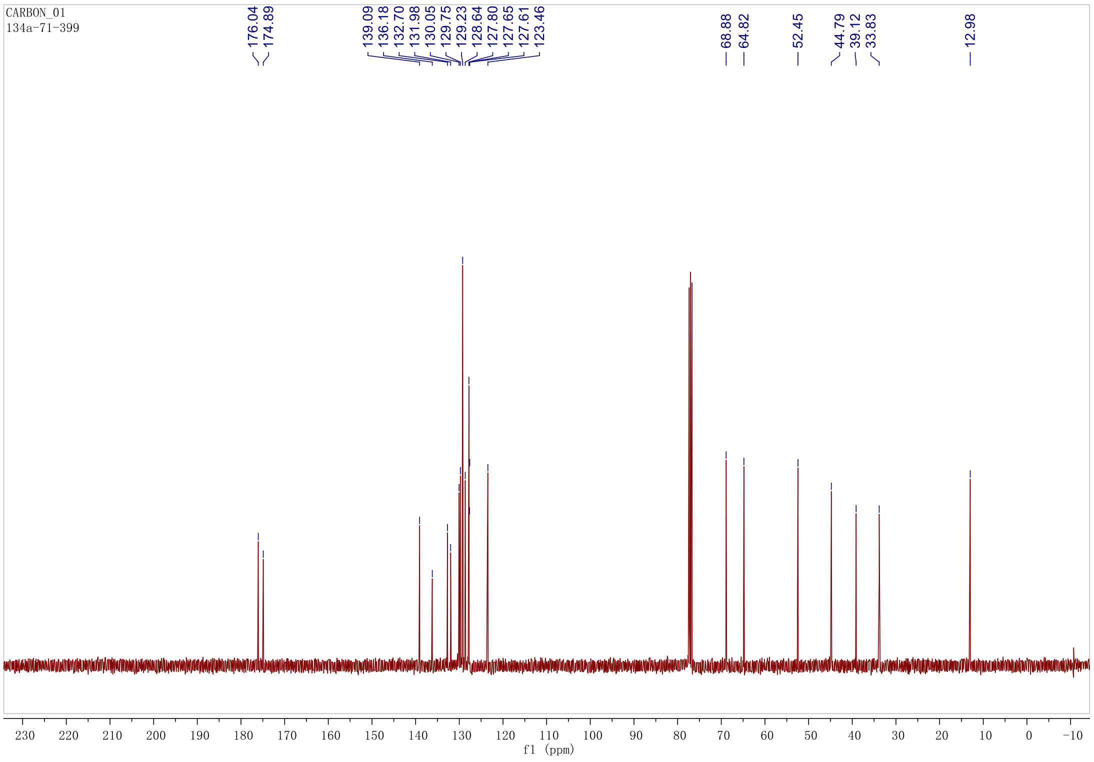
CARBON_01
134a-71-399
176.04
174.89
139.09
136.18
132.70
131.98
130.05
129.75
129.23
128.64
127.80
127.65
127.61
123.46
68.88
64.82
52.45
44.79
39.12
33.83
12.98
f1 (ppm)

PROTON_01
134a-41-478

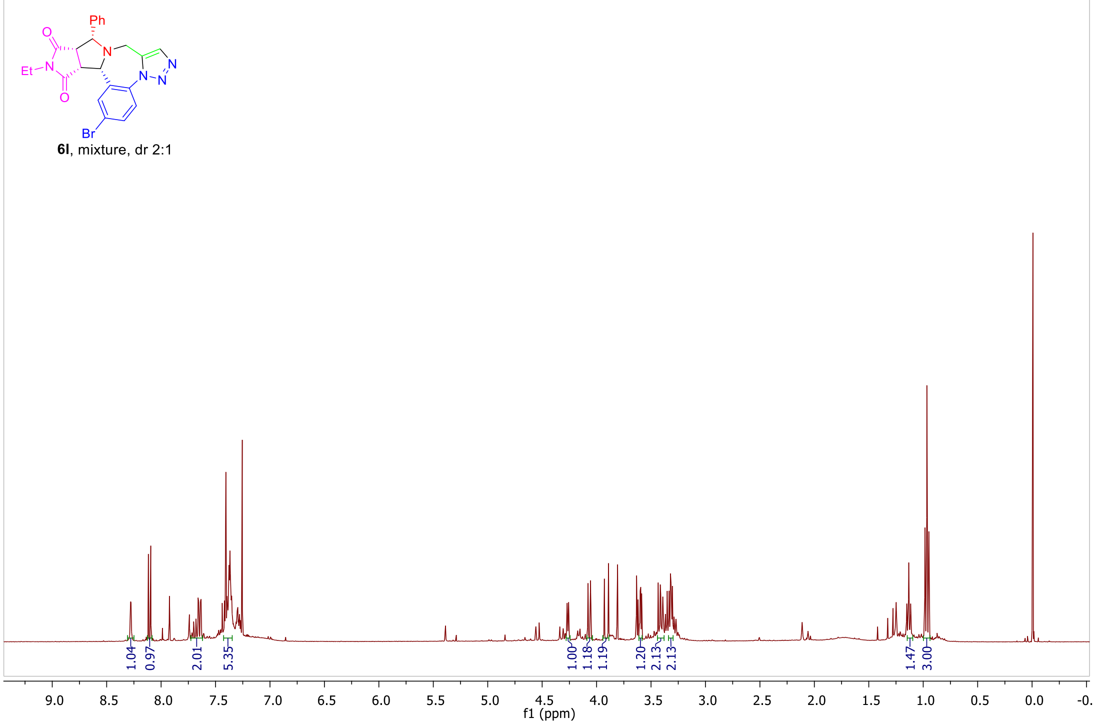

6l, mixture, dr 2:1

CARBON_01
134a-41-478

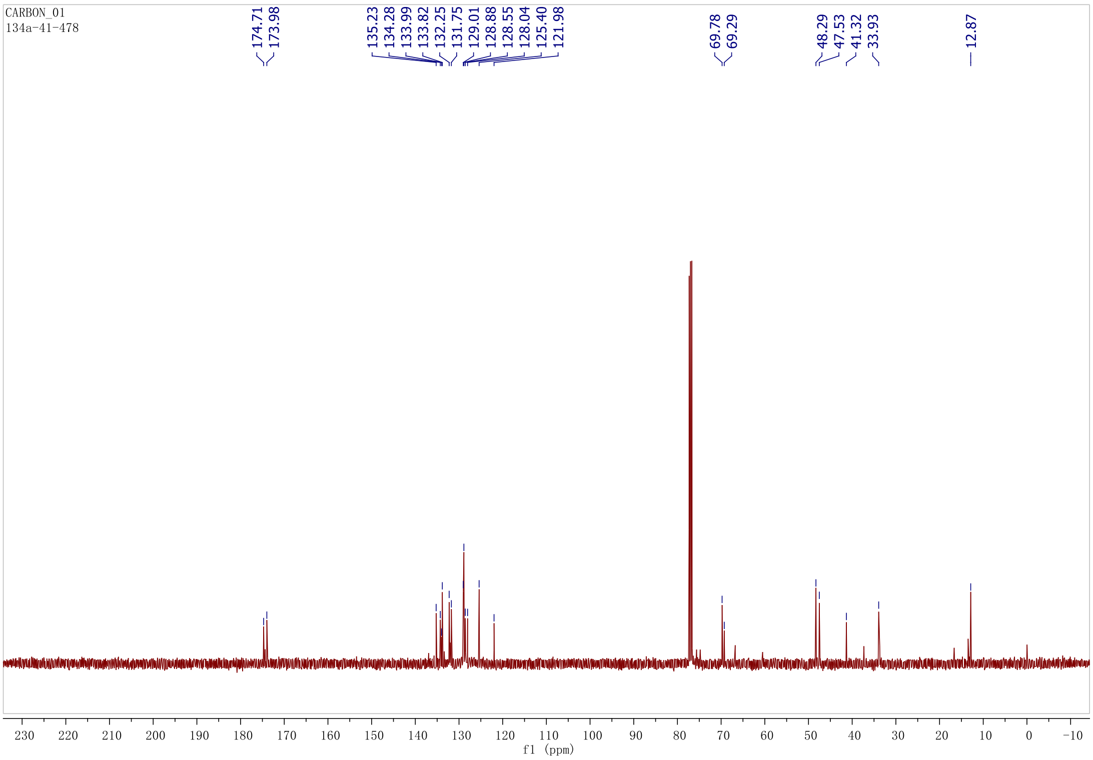

Supplement: Supplementary file 1 [file molecules-24-00601-s001.pdf]
